# A Quantum Chemical Method for Dissecting London Dispersion Energy into Atomic Building Blocks

**DOI:** 10.1021/acscentsci.5c00356

**Published:** 2025-05-08

**Authors:** Gianluca Regni, Lorenzo Baldinelli, Giovanni Bistoni

**Affiliations:** Department of Chemistry, Biology and Biotechnology, University of Perugia, Via Elce di Sotto, 8 06123 Perugia, Italy

## Abstract

London dispersion (LD) forces are ubiquitous in chemistry and biology, governing processes such as binding of drugs to protein targets, the formation and stability of reaction intermediates, and the selectivity of enantioselective transformations. Developing an experimental or quantum chemical method to quantify atomic contributions to LD energy could open up new pathways for controlling reaction selectivity and guiding molecular design. Herein, we initially introduce Atomic Decomposition of London Dispersion energy (ADLD), a computational method that provides atomic-level resolution in quantifying LD energy at the “gold standard” level of quantum chemistry. Through a series of case studies, we reveal that LD is highly sensitive to variations in the electronic structure, including spin state, charge, and valence bond resonance effects—key factors often overlooked. Furthermore, we uncover the fundamental origin of the recently proposed *gravitational-like* relationship describing the distance dependence of LD energy in molecular systems. In doing so, we reconcile these recent findings with Fritz London’s original formulation in 1930, offering a unified perspective on the fundamental nature of LD forces.

## Graphical abstract

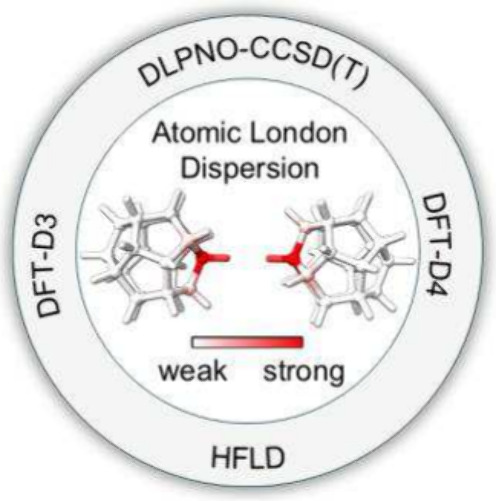

## 1 Introduction

London dispersion forces, first described by London in 1930,^1,2^ are fundamental to a wide range of chemical and biological processes, influencing molecular aggregation in the gas phase as well as the behavior of complex systems in solution. These long-range interactions are pivotal in diverse phenomena, from catalytic selectivity and protein folding to the stability of molecular crystals and the reactivity of complex materials.^3–7^

A major challenge in the study of dispersion interactions lies in obtaining detailed insights into the contribution of each functional group, or even individual atoms, to their strength. Specifically, what is the contribution of a given atom or functional group to the dispersion energy of a system, to the dispersion interaction between two monomers, or to the stabilization of a transition state and, hence, to the kinetics of a given reaction channel? Such detailed insights are crucial for enabling the design of new materials, drugs, and catalysts with tailored properties.

Motivated by this need, our research group recently introduced the Atomic Decomposition of London Dispersion energy (ADLD) method, which allows for the quantification of dispersion contributions from individual atoms within a molecular system.^8^ The first implementation of this approach, based on cost-effective mean-field methods, provides a qualitatively accurate representation of atomic contributions to London dispersion energy and has already found widespread applications in molecular chemistry.

However, achieving a complete understanding of London dispersion forces, and effectively harnessing them for chemical applications, requires overcoming a key limitation of mainstream computational methodologies based on semiclassical dispersion corrections: accurately accounting for the influence of electronic structure effects—such as spin, resonance, charge, and many-body interactions—on the atomic dispersion energy. For example, recent computational studies have revealed a *gravitational-like* dependence of London dispersion energy on molecular masses and distances for a series of molecular dimers.^9^ This was attributed to enhanced contributions of oscillating-ionic valence bond structures that propagate charges across molecular frameworks. More generally, electronic effects can greatly influence the strength and nature of dispersion forces, and capturing them with high accuracy is essential for understanding intermolecular interactions in their full complexity.^10–13^

In this work, we preset a computational method that accurately quantifies the contribution of each atom to the dispersion energy by combining the ADLD scheme with the Local Energy Decomposition (LED) framework^14,15^ at the domain-based local pair natural orbital coupled cluster DLPNO-CCSD­(T) level.^16–18^ This approach exploits the local nature of electron correlation to disentangle the dispersion energy from other correlation effects. Since this approach is based on the “gold standard” coupled-cluster theory,^19^ it inherently accounts for the intricate interplay of electronic structure effects on dispersion interactions.

The ADLD/LED scheme is then used to gain atomic-level insights into commonly observed yet usually overlooked London dispersion effects, focusing on quantifying phenomena closely tied to changes in the electronic structure of the chemical system under study. The results are then compared with those obtained by integrating ADLD with Grimme’s D4 dispersion model, through which many-body and electronic structure effects can be incorporated in an approximate yet effective way.^10^

Specifically, we first examine the influence of spin and charge effects on dispersion using the ionization of doublet C_6_H_6_–Li to singlet C_6_H_6_–Li^+^ as an illustrative case study. Next, we apply our method to quantify the influence of resonance effects on the dispersion contributions governing the relative stability of two isomers of a recently developed molecular balance, designed for experimentally determining London dispersion effects in solution. For this purpose, we examine the isomerization of cyclooctatetraene (COT) substituted with alkyl groups in the 1,4- and 1,6-positions. These two isomers can be interconverted to each other via valence-bond isomerization exhibiting a high sensitivity to environmental effects.^20^ Finally, our analysis elucidates the underlying mechanisms responsible for the *gravitational-like* relationship observed in dispersion interactions, reconciling these findings with Fritz London’s original description of London dispersion forces from 1930.^9^

## 2 Results and Discussion

### 2.1 The ADLD Scheme

In the present work, the ADLD scheme is employed to quantify atomic dispersion contributions obtained using three different methodologies: the semiclassical D3 ^21,22^ and D4 ^10^ corrections at the DFT level, and the LED scheme at the DLPNO-CCSD­(T) level. While the decomposition of semiclassical corrections was discussed in the original ADLD paper^8^ (see Section S4 in the Supporting Information for further details), the atomic decomposition at the LED/DLPNO-CCSD­(T) level is presented here for the first time, warranting a more in-depth discussion.

To begin, we note that London dispersion is a long-range dynamic correlation effect.^23^ For post-HF methods, the correlation energy (*E*_*C*_) can be expressed as a sum of pair correlation energies

$$E_{C}=\underset{i>j}{\sum }\varepsilon_{ij}$$

where *i* and *j* denote the occupied spin orbitals.

In systems held together by noncovalent interactions, the dominant contribution to the correlation binding energy is typically London dispersion.^24–27^ Consequently, an approximate estimate of atomic dispersion energies was proposed in ref 8 by directly decomposing the correlation energy. However, more generally, *E*_*C*_ incorporates both long-range and short-range electron correlation effects. To disentangle the different components of the correlation energy and isolate a “pure” London dispersion energy (*E*_*disp*_), the LED scheme can be used. Specifically, pair correlation energies can be written as a sum of double excitation contributions

$$\varepsilon_{ij}=\underset{{\overset{\sim }{a}}_{ij}{\overset{\sim }{b}}_{ij}}{\sum }\left(i{ã}_{ij}|j{\mathrm{b̃}}_{ij}\right){\mathrm{\tau ̃}}_{{ã}_{ij}{\mathrm{b̃}}_{ij}}^{ij}$$

where *ã*_*ij*_ and *b̃*_*ij*_ are Pair Natural Orbitals (PNOs) belonging to the pair of occupied orbitals *ij*; (*iã*_*ij*_|*jb̃*_*ij*_) terms are the two-electron integrals; *τ̃*_*ã*_*ij*_ *b̃*_*ij*__^*ij*^ are the contravariant cluster amplitudes. By exploiting the local nature of occupied and virtual orbital spaces in the DLPNO framework and assigning each orbital to the fragment in which it is dominantly localized, this approach allows us to achieve a precise quantification of the London dispersion energy. By assigning PNOs onto fragments, *ε*_*ij*_ can be partitioned into different families of double excitation contributions, as shown graphically in Figure 1. Accordingly, pair dispersion energies can be defined as

$$\varepsilon_{ij}^{disp}=\underset{{\overset{\sim }{a}}_{X}{\overset{\sim }{b}}_{Y}}{\sum }\left(i_{X}{ã}_{X}|j_{Y}{\mathrm{b̃}}_{Y}\right){\mathrm{\tau ̃}}_{{ã}_{X}{\mathrm{b̃}}_{Y}}^{i_{X}{\;j}_{Y}}$$

where the subscripts *X* and *Y* are used to identify the fragments in which the orbitals are localized. The total dispersion energy can thus be written as

$$E_{disp}=\underset{i>j}{\sum }\varepsilon_{ij}^{disp}$$

By assigning half of the pair dispersion energy contribution to each electron, dispersion energy contributions of single electrons *ε*_*i*_^*disp*^ are obtained

$$E_{disp}=\underset{i>j}{\sum }\varepsilon_{ij}^{disp}=\frac{1}{2}\underset{i}{\sum }\underset{j\neq i}{\sum }\varepsilon_{ij}^{disp}=\underset{i}{\sum }\varepsilon_{i}^{disp}$$

**Figure 1:**
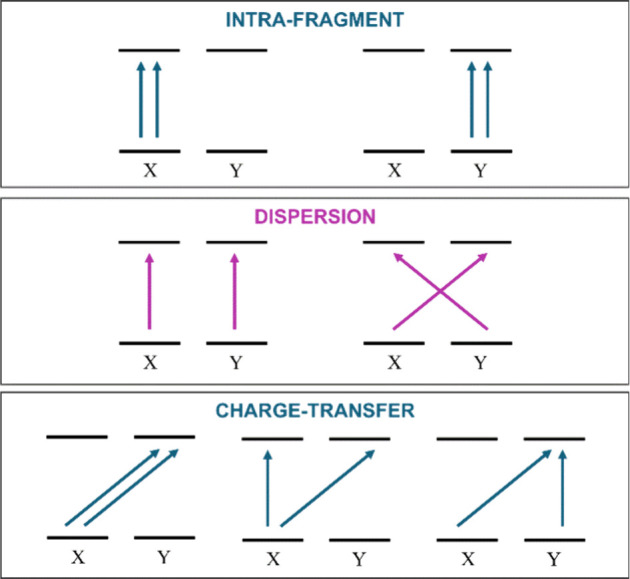
Illustration of the different families of double excitations from occupied to virtual orbitals that contribute to the correlation energy in the LED scheme. For simplicity, only CT excitations from fragment X to Y are shown. Dispersion excitations are highlighted in purple.

To define the atomic dispersion contributions *ε*_*A*_^*disp*^, a charge partition scheme that maps each *ε*_*i*_^*disp*^ onto individual atoms is required

$$E_{C}=\underset{i}{\sum }\varepsilon_{i}^{disp}=\underset{A}{\sum }\underset{i}{\sum }\omega_{Ai}\varepsilon_{i}^{disp}=\underset{A}{\sum }\varepsilon_{A}^{disp}$$

where ω_*Ai*_ represents the fraction of electronic charge for orbital *i* assigned to atom *A*. A similar decomposition for the triples correction is detailed in Section S5 in the Supporting Information. We note here that an alternative way to project observables into arbitrarily defined fragments has been recently proposed by Gori, Kurian and Tkatchenko in the many-body dispersion framework.^28^

It is important to emphasize here that, in the ADLD framework, atomic contributions depend on the chosen population scheme. However, the influence of the population scheme is minimal for the decomposition of the dispersion energy, as detailed in the Supporting Information (see Table S11, Figures S19 and S20). In addition, the atomic dispersion contributions show smooth convergence by increasing the basis set size (see Table S11, Figures S19 and S20).

A London dispersion density ρ_*disp*_, a function of spatial coordinates *r*, can be defined to easily visualize and analyze the atomic contributions

$$\rho_{disp}\left(r\right)={\left(\frac{\pi }{\alpha }\right)}^{-3/2}\underset{A}{\sum }\varepsilon_{A}^{disp}e^{-\alpha (r-R_{A}{)}^{2}}$$

where α is a parameter that needs to be adjusted for visualization purpose (typically set to 0.5) and *R*_*A*_ is the atom position. Since we typically focus on relative energies rather than total energies, such as in reactivity or molecular recognition investigations, a London dispersion difference density function Δρ_*disp*_ can also be defined

$${\Delta \rho }_{disp}\left(r\right)={\left(\frac{\pi }{\alpha }\right)}^{-3/2}\underset{A}{\sum }\Delta \varepsilon_{A}^{disp}e^{-\alpha (r-R_{A}{)}^{2}}$$

where Δ*ε*_*A*_^*disp*^ is the difference between the atomic London dispersion energy of atom *A* for two different molecular structures. Clearly, the use of this function requires a one-to-one mapping between the atoms of the two structures (e.g., reactant and products in an elementary process). This approach can be particularly useful for rationalizing dispersion contributions from specific functional groups to reaction pathways or conformational equilibria.

### 2.2 Computational Details

All calculations were performed with a development version of ORCA quantum chemistry package based on version 6.1.^29,30^ Unless otherwise specified, all calculations were carried out at the DLPNO-CCSD­(T)^16–18^ level in conjunction with the LED^15,31,32^ scheme for the ADLD. Foster-Boys localization^33^ scheme and Löwdin population analysis were employed. DFT calculations were carried out including Grimme’s D3­(BJ) and/or D4 dispersion corrections.^21,22^ Ahlrichs’ def2-QZVP basis set was utilized, while cc-pVTZ basis set was employed exclusively in Section 2.3.3. In the following, the ADLD scheme is denoted as ADLD­(LED), ADLD­(D3), and ADLD­(D4) when applied at the DLPNO-CCSD­(T)/LED, D3, and D4 levels, respectively. See Section S1 in Supporting Information for additional details. The tools for computing the ρ_*disp*_ and Δρ_*disp*_ are available freely online.^34^ Geometry optimization of the C_6_H_6_–Li system was carried out at the DFT level using the B3LYP functional, combined with Grimme’s D4 dispersion correction and the def2-TZVP basis set. To ensure consistency with the geometries of the other dimers taken from ref 9, the geometries of the newly included dimers were optimized at the PBE0-D4/cc-pVTZ level.

### 2.3 Case Studies

In this section, we provide a quantitative analysis of various electronic structure effects influencing the magnitude of the London dispersion energy, as well as their influence on experimental observables like binding energies. For this study, unless otherwise specified, we employed the newly developed ADLD­(LED) scheme.

We began by examining the influence of spin and charge effects on atomic dispersion contributions, using the interaction between benzene and a lithium atom as a case study. Next, we explored the impact of resonance effects on the equilibrium between two isomers of a COT substituted with alkyl groups in the 1,4- and 1,6-positions across different environments. Finally, we elucidated the underlying origins of the recently proposed *gravitational-like* relationship observed in dispersion interactions within molecular dimers.^9^

#### 2.3.1 Analysis of Charge and Spin Effects

When comparing the interactions of doublet Li and singlet Li^+^ with other molecules, one would expect the dispersion interaction involving Li to be significantly stronger than that of Li^+^ for the same geometry, due to the stark difference in their polarizabilities. Specifically, ongoing from Li to Li^+^, the experimental polarizability decreases by 24.3 Å^3^ (99.9%).^35–37^ As an illustrative example of how this effect is captured by the proposed model, we investigated the interaction of Li with benzene (Figure 2).

**Figure 2:**
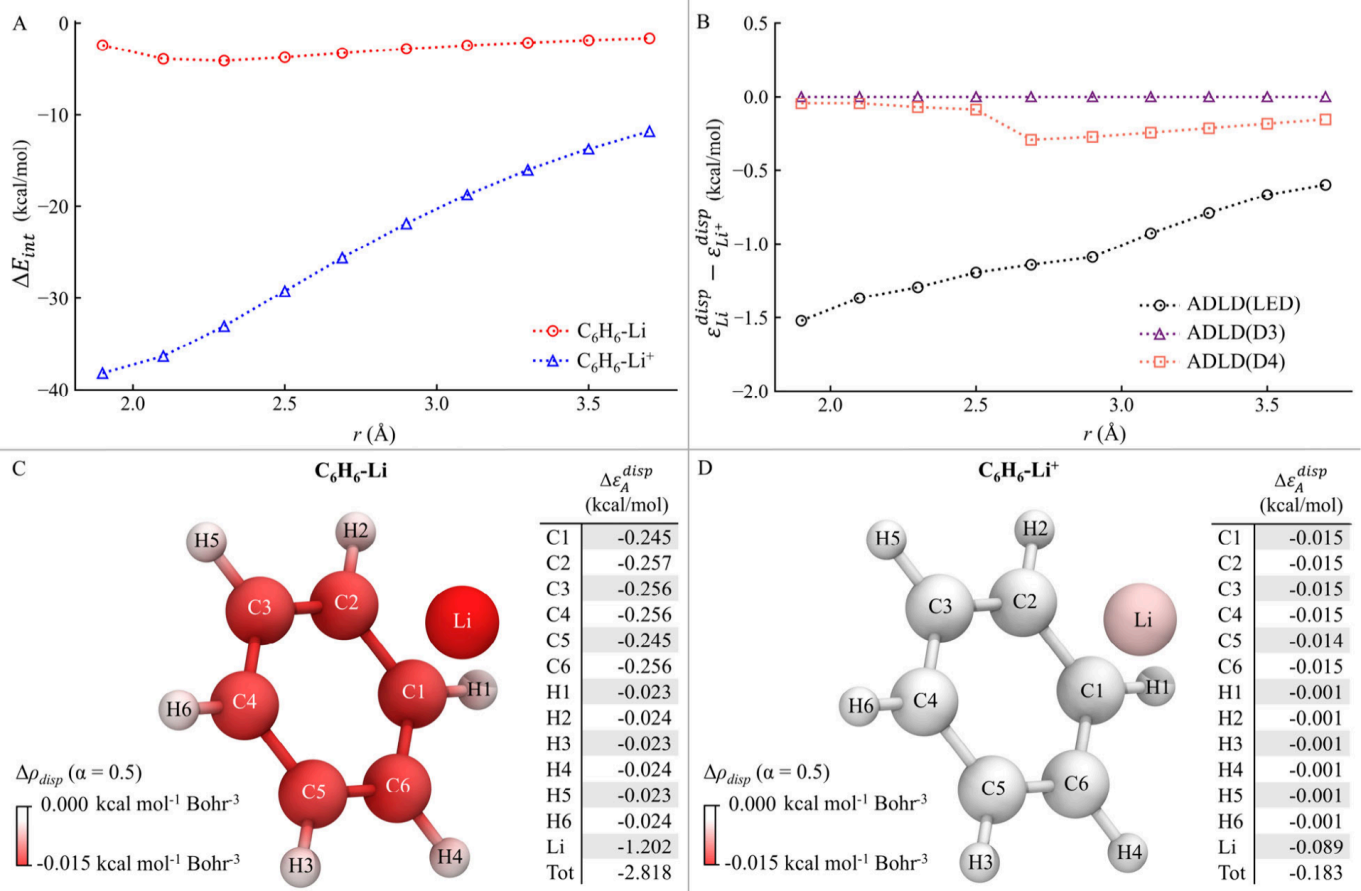
(A) C_6_H_6_···Li and C_6_H_6_···Li^+^ interaction energies (Δ*E*_*int*_) as a function of the distance (*r*) between the Li atom and the center of mass of the benzene ring, computed at the DLPNO-CCSD­(T) level. (B) Differences in the atomic dispersion contributions (*ε* ^*disp*^) of Li and Li^+^ atoms in a benzene-lithium complex as a function of *r*, computed at the ADLD­(LED), ADLD­(D3) and ADLD­(D4) levels. (C, D) ADLD­(LED) dispersion density difference function Δρ_*disp*_ (α = 0.5) alongside the corresponding Δ*ε*_*A*_^*disp*^ for C_6_H_6_–Li and C_6_H_6_–Li^+^ at *r* = 2.69Å.

The decay of the C_6_H_6_···Li and C_6_H_6_···Li^+^ interaction energies with the distance *r* between Li atom and the center of mass of benzene ring for C_6_H_6_–Li and C_6_H_6_–Li^+^ is reported in Figure 2A. The positively charged system exhibits a significantly higher interaction at all distances, primarily due to electrostatic forces. The London dispersion interaction between the two fragments is only a small portion of the total correlation energy.^26^ Therefore, decoupling the various contributions is essential to accurately isolate and evaluate the London dispersion component.

Figure 2B illustrates the difference in the London dispersion energy contributions of the Li atoms in its neutral and +1 charge states as a function of *r*. As expected, the D3 method shows no sensitivity to changes in the system charge at any distance. This limitation arises from the inherent assumption in the D3 model that the dispersion energy depends solely on geometry and remains independent of the electronic structure.

In contrast, the D4 method incorporates a more advanced treatment of dispersion, capturing variations in atomic dispersion contributions as the charge state changes. For example, at the equilibrium geometry, the London dispersion contribution of the Li atom decreases by 0.29 kcal/mol when transitioning from the neutral to the positively charged system. The “bump” observed in the D4 curve at short distances is attributed to abrupt changes in the C_6_ coefficients along *r*.^38,39^

While methods like D4 and other modern semiclassical correction schemes effectively account for charge effects, London dispersion forces originate from long-range dynamic electronic correlation. As such, highly correlated wave function-based approaches, such as DLPNO-CCSD­(T)/LED, remain the preferred methods for studying intricate electronic structure effects on dispersion energy. At the C_6_H_6_–Li equilibrium geometry, ADLD­(LED) reveals a decrease of 1.03 kcal/mol in the atomic dispersion contribution of the Li atom when moving from the neutral to the positively charged system. Overall, the qualitative trends observed at the D4 level align with those obtained from the coupled-cluster approach. However, the effect captured by DLPNO-CCSD­(T) is more pronounced and varies more smoothly with *r*.

Figure 2C and Figure 2D display Δρ_*disp*_(*r*) and the corresponding atomic decomposition of the London dispersion energy for the benzene-lithium interaction in its neutral and +1 charge states, respectively, at the ADLD­(LED) level. These visualizations provide a striking representation of how the positively charged system exhibits consistently lower atomic contributions to London dispersion across all atoms compared to the neutral system. The atomic decomposition further allows for a precise quantification of changes in the contribution from each atom. Notably, the contribution from the Li atom to the dispersion interaction decreases significantly by approximately 1.1 kcal/mol (−92.5%) when transitioning from the neutral to the positively charged system. This analysis highlights the utility ADLD­(LED) in capturing and quantifying subtle changes in dispersion interactions arising from variations in electronic structure.

#### 2.3.2 Resonance Effects

The equilibrium between 1,6-di*tert*butyl-COT (***t*** **Bu1,6**) and 1,4-di*tert*butyl-COT (***t*** **Bu1,4**) is governed by intramolecular London dispersion forces, making the sterically more crowded ***t*** **Bu1,6** more stable than the ***t*** **Bu1,4**, both in gas phase and in a wide range of solvents.^20^ The main chemical mechanism behind this effect has already been investigated in previous works. Specifically, previous analysis suggested that the key dispersion interactions operating in this system can be categorized as σ–σ and σ–π dispersion interactions^8^ (more information is provided in Section S2.2).

The subtle variations in the dispersion contributions of individual atoms determine the relative stability between the isomers. Qualitatively, a first insight into this aspect can be obtained by analyzing the Δρ_*disp*_ between the two isomers (Figure 3C). A red region around the two methyl groups of *tert*-butyl substituents confirms that σ–σ CH–CH dispersion interactions (Figure 3A) contribute to the greater stability of more crowded isomer.

**Figure 3:**
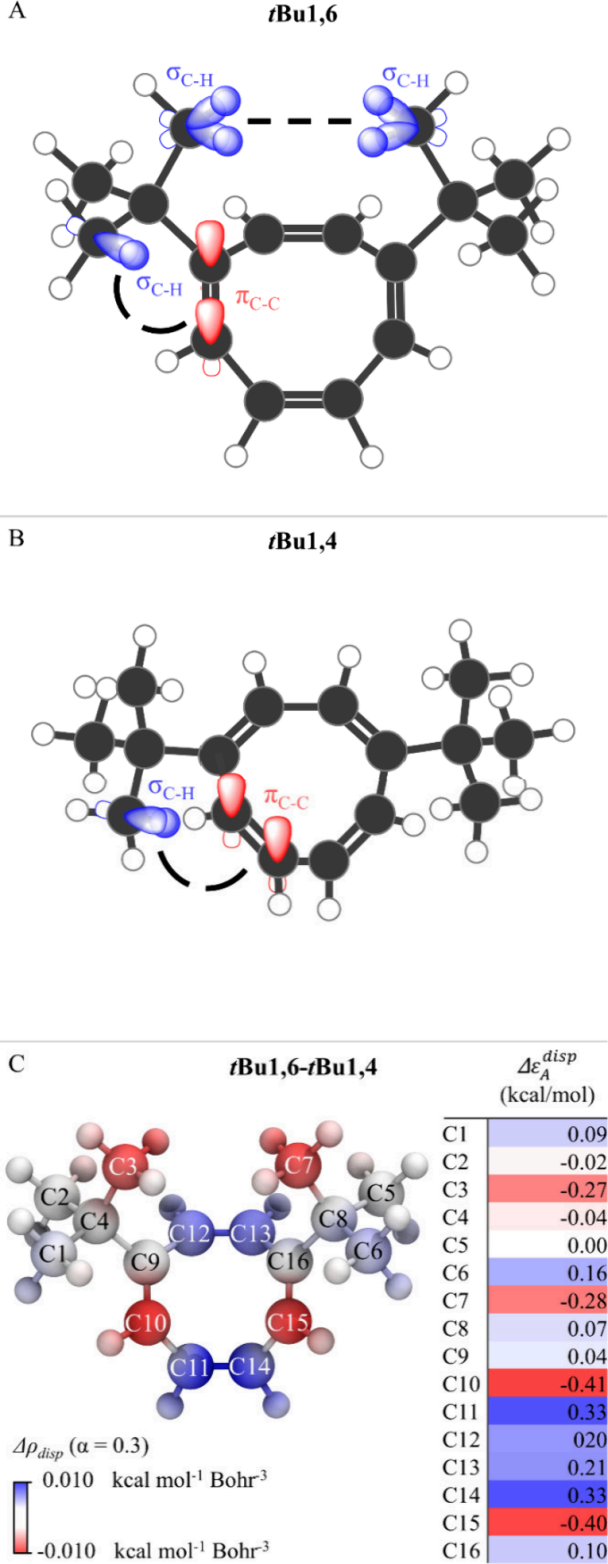
σ–σ and σ–π dispersion interactions in ***t*** **Bu1,6** (A) and ***t*** **Bu1,4** (B). (C) The ADLD­(LED) dispersion density difference function Δρ_*disp*_ (α = 0.3) with the corresponding Δ*ε*_*A*_^*disp*^ for ***t*** **Bu1,6**-***t*** **Bu1,4** (hydrogens are not shown for simplicity; the two *tert*-butyl groups and the central ring are defined as fragments in the LED calculations).

The analysis also reveals varying contributions to this differential stability, both positive and negative, from the central ring carbons, which are attributed to σ–π dispersion interactions between the hydrogen atoms of the *tert*-butyl groups and the double bond of the central ring (Figure 3A and Figure 3B). These interactions become stronger as the σ–π distances decrease. As the system transitions from ***t*** **Bu1,6** to ***t*** **Bu1,4**, the position of the double bond shifts (see Figure S9), altering the relative contributions of the ring atoms to the overall dispersion energy. Quantitatively, the total contributions from the *tert*-butyl groups and the central ring, calculated by summing the respective atomic contributions, are −0.55 and +0.42 kcal/mol, respectively. These results suggest that the σ–σ CH–CH interactions are predominantly responsible for the experimentally observed differential stability between the isomers.

This case study highlights the in-depth level of insight that ADLD­(LED) can provide in determining the relative importance of individual atoms to the *intramolecular* dispersion energy of a chemical system. Even in situations involving intricate electronic structure effects, such as resonance effects, this approach offers a powerful framework to analyze dispersion contributions to properties such as conformational preferences across various environments.

It is also of interest to discuss how the ADLD­(LED) tool can be used to analyze the complex pattern of *intermolecular* dispersion interactions contributing to phase stability in apolar molecular crystals. To this end, we examined the solid-state structure of 1,4-diadamantyl-cycloocta­tetraene (1,4-di-Ad-COT), as determined experimentally.^20,40^ In the present case, our model system consists of a cluster of 11 monomers extracted from the experimental X-ray crystal structure (Figure 4A).

**Figure 4:**
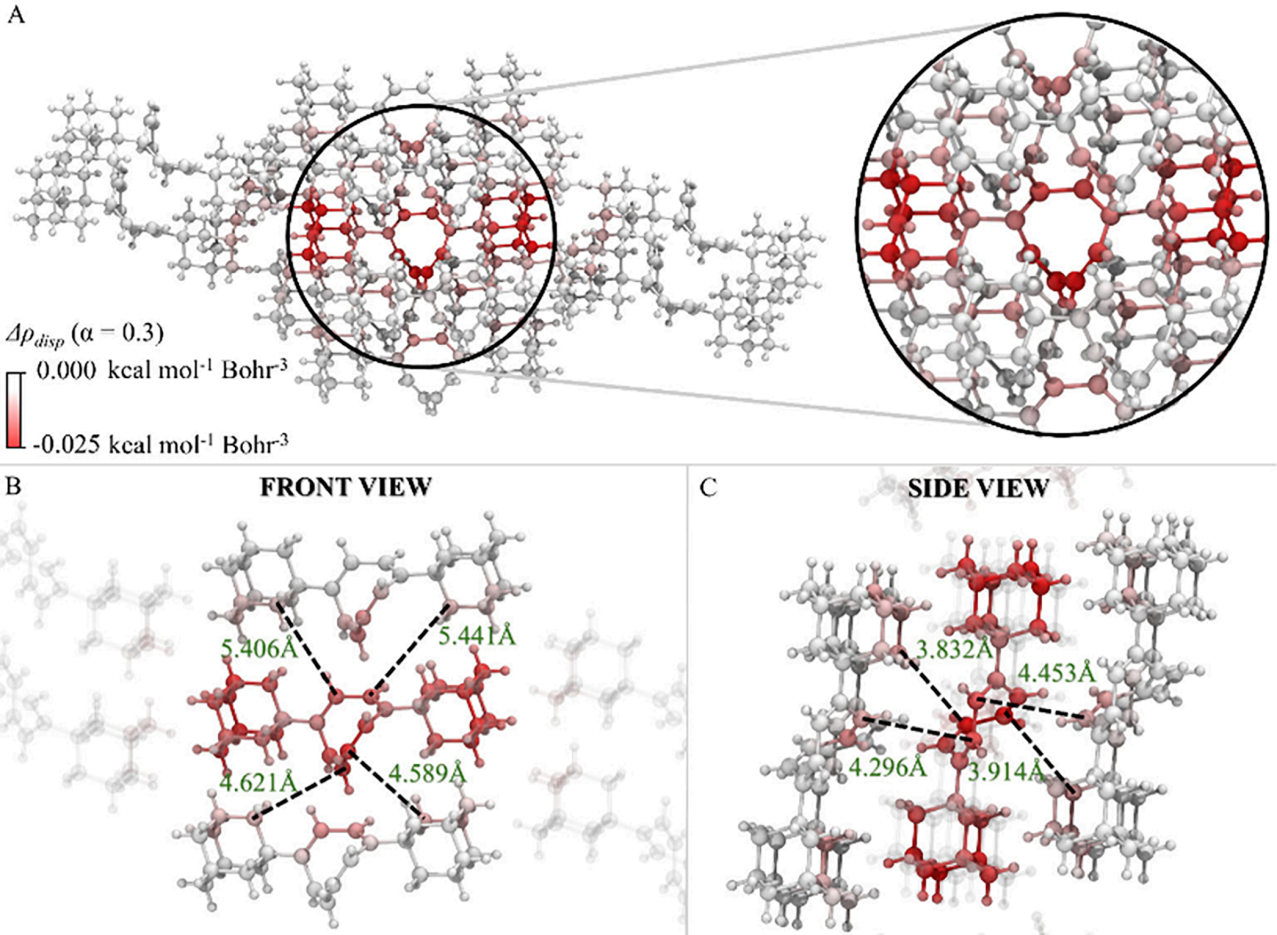
Δρ_*disp*_ (α = 0.3) associated with the interaction of the central monomer with its surrounding monomers in a cluster model of the 1,4-di-Ad-COT crystal. Different views are shown: (A) zoom-in on the central monomer, (B) front view, and (C) side view. Calculations were performed at the DLPNO-CCSD level using Löwdin population analysis. The central monomer and the surrounding monomers are defined as fragments.

In our previous work,^40^ it was observed that the lattice energy in this system is dominated by London dispersion interactions. The dispersion density difference plot (Figure 4A) provides a spatial analysis of the dispersion interactions between the central monomer and its surrounding monomers with atomic resolution. Remarkably enough, the Δρ_*disp*_ map shown in Figure 4A reveals a relatively uniform distribution of dispersion contributions among the carbon atoms of the central monomer.

Quantitatively, each carbon atom contributes a stabilizing dispersion energy ranging from 0.5 to 0.9 kcal/mol to the lattice energy, as detailed in Section S2.3 of Supporting Information.

These results illustrate that the molecular packing in the solid-state is optimized to maximize dispersion interactions across all atoms. Such packing arrangements, driven by dispersion forces, are a common feature for apolar molecules in the condensed phase, and hence the ADLD scheme appears as a powerful tool to analyze the key forces that govern crystal assembly.

#### 2.3.3 Analysis of *Gravitational-like* Relationship of London Dispersion

Recently, Shaik and co-workers proposed a *gravitational-like* dependence for London dispersion interactions in molecular systems.^9^ Specifically, they demonstrated for a set of dimers that the total London dispersion energy is proportional to the product of the two molecular masses (*M*_1_ *·M*_2_) and inversely proportional to the distance (*R*) between their centers of mass. Using the ADLD scheme, we investigated the nature of this relationship. We analyzed a limited but representative set of dimers, illustrated in Figure 5A. **1**–**5** were selected from the original work of Shaik and co-workers, while **6**–**9** were examined here for the first time (details provided in Section S2.4 in the Supporting Information). Figure 5B confirms the linear correlation between the total London dispersion energy *E*_*tot*_^*disp*^ and *M*_1_ *·M*_2_/*R* for **1**–**5** (R^2^ = 0.995). However, **6**–**8** clearly deviate from this correlation, and their inclusion significantly reduces the quality of the linear fit (R^2^ = 0.742). To understand the origin of this behavior, we decomposed *E*_*tot*_^*disp*^ into contributions associated with *intramolecular* and *intermolecular* dispersion forces. Within a supramolecular approach, *E*_*tot*_^*disp*^ can be exactly written as a sum of isolated monomers contributions (*E*_1_^*disp*^ + *E*_2_^*disp*^), plus a contribution representing the interaction between the monomers (*E*_*int*_^*disp*^):

$$E_{tot}^{disp}={E_{1}^{disp}+E_{2}^{disp}+E}_{\;\mathrm{int}}^{disp}$$

The intramolecular dispersion terms, *E*_1_^*disp*^ and *E*_2_^*disp*^, are calculated from the isolated monomers and are independent of *R*. Hence, for the linear correlation between *E*_*tot*_^*disp*^ and *M*_1_ *·M*_2_/*R* to be generally valid, the contribution to *E*_*tot*_^*disp*^ from the intramolecular dispersion terms should be negligible when *R* varies significantly.

**Figure 5:**
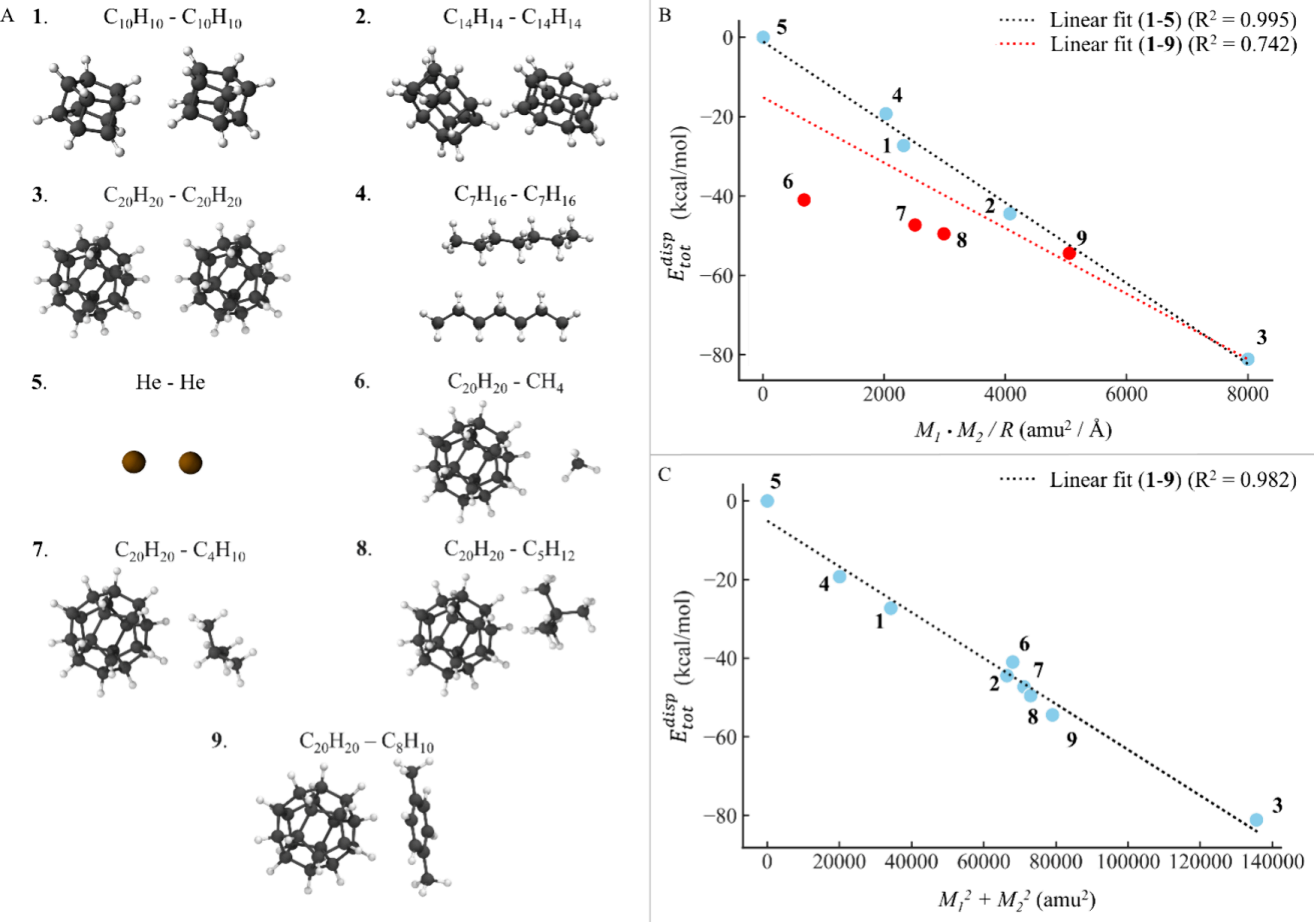
(A) Set of dimers used for the study. (B) Dependence of total dispersion energy (*E*_*tot*_^*disp*^) at the PBE0-D4 level on (*M*_1_ *·M*_2_ */R*), where *M*_1_ and *M*_2_ are the molecular masses of the dimer and *R* is the distance between the respective centers of mass. (C) Dependence of total dispersion energy (*E*_*tot*_^*disp*^) at the PBE0-D4 level on (*M*_1_^2^ + *M*_2_^2^), where *M*_1_ and *M*_2_ are the molecular masses of the dimer.

In contrast, *E*_*tot*_^*disp*^ is typically dominated by *E*_1_^*disp*^ and *E*_2_^*disp*^, because intramolecular dispersion forces usually outweigh intermolecular forces. Furthermore, *E*_*int*_^*disp*^ does not show any correlation with *M*_1_ *·M*_2_/*R* (R^2^ = 0.187, see Figure S12). Hence, the observed *gravitational-like* relationship for **1**–**5** is only possible because *R* does not vary significantly within the considered set of dimers, and because the influence of intermolecular dispersion forces on the total dispersion energy is minimal. Accordingly, a linear correlation of similar quality is also expected between the intramolecular dispersion energy (*E*_1_^*disp*^ + *E*_2_^*disp*^) and *M*_1_ *·M*_2_. Indeed, these two quantities are correlated with an R^2^ value of 0.991 and 0.713 for **1**–**5** and **1**–**9**, respectively (see Figure S13).

To explain this relationship, consider the intramolecular dispersion energy for a monomer, assuming atom-pairwise additivity

$$E_{1}^{disp}\propto \underset{A,B}{\sum }\frac{\alpha_{A}\alpha_{B}}{{r_{AB}}^{6}}$$

where α_*A*_ and α_*B*_ are the atomic polarizabilities, and *r*_*AB*_ is the distance between the atom pair. Since the mass of a neutral atom is generally proportional to its polarizability, *M*_1_ ∝ ∑_*A*_ α_*A*_, where α_*A*_ is the polarizability of atom *A* in the monomer. In addition, if the system is relatively compact, such that *r*_*AB*_ varies within a narrow range, we can approximate *r*_*AB*_^6^ as a constant. Thus, we arrive at an approximate expression for *E*_1_^*disp*^:

$$E_{1}^{disp}\propto {M_{1}}^{2}$$

By extending this reasoning to the second monomer, and neglecting intermolecular dispersion forces, one arrives at the following approximate expression

$$E_{tot}^{disp}\propto {M_{1}}^{2}+{M_{2}}^{2}$$

from which it is possible to derive a generalized equation describing the dependence of the total dispersion energy on the molecular masses:

$$E_{tot}^{disp}=\beta \left({M_{1}}^{2}+{M_{2}}^{2}\right)+\gamma$$

where β has the unit of $\frac{\mathrm{kcal}}{\mathrm{mol}\;{\mathrm{amu}}^{2}}$ and γ has the unit of $\frac{\mathrm{kcal}}{\mathrm{mol}}$ and incorporates the average effect of intermolecular dispersion interactions. This *generalized* expression is valid for the dimers considered here, with an R^2^ value for **1**–**9** of 0.982 (β = 5.8216 × 10^–4^, γ = −5.0667), the highest correlation achieved (Figure 5C). Extending this analysis to the full dimer set proposed by Shaik maintains high accuracy, leading to an R^2^ value of 0.974. The *gravitational-like* dependence for **1**–**5** in Figure 5B and for the entire original set, arises because the monomers in all dimers have identical or very similar masses. Indeed, when *M*_1_ = *M*_2_ we have that *M*_1_^2^ + *M*_2_^2^ = 2*M*_1_ *M*_2_, and hence the *generalized* relationship essentially simplifies to the expression originally proposed by Shaik, aside for the irrelevant distance dependence. When dimers consisting of different monomers are used, such as **6**–**9**, the *gravitational-like* relationship breaks (Figure 5B), while the *generalized* expression provided here still holds (Figure 5C). It is worth noting here that, as the system grows, *E*_1_^*disp*^ is increasingly dominated by short-range interactions between neighboring atoms, whose number scales linearly with the number of atoms rather than quadratically. Thus, the total dispersion energy of the systems correlates better with the sum of the masses of the dimer (Figure S15).

Finally, the observation that intermolecular dispersion forces are not directly related in any simple way to the masses of the interacting monomers deserves to be discussed in more detail. Figure 6 shows the dispersion density difference function for the interaction between two dodecahedrane molecules at their dimer equilibrium geometry, computed at the ADLD­(D4) and ADLD­(LED) levels. Notably, the interaction is governed by a limited set of atoms at the contact region. Increasing the masses of the dimers by including additional atoms far from the contact point does not significantly affect the intermolecular dispersion energy. Indeed, **6** was derived by removing 19 of the 20 carbon atoms from one dodecahedrane molecule and reoptimizing the structure, yet it still exhibits a noticeable dispersion energy. While this energy is attenuated compared to **3**, it remains significant. These findings underscore the utility of the ADLD­(LED) scheme introduced here for analyzing the spatial origin of intermolecular dispersion forces. Applications of this approach to experimentally relevant systems where dispersion plays a key stabilizing role are currently underway.^41,42^

**Figure 6:**
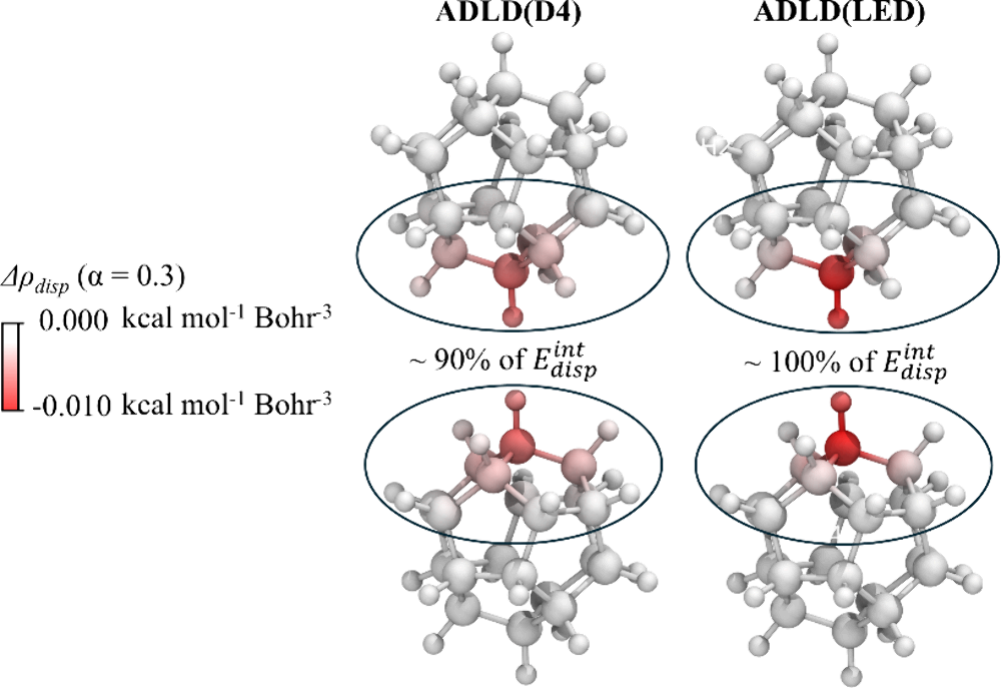
Dispersion density function ρ_disp_ (α = 0.3) of dodecahedrane dimer^9^ dispersion interaction at the ADLD­(D4) and ADLD­(LED) levels.

## 3 Conclusions

We presented a powerful quantum chemical approach to quantify the contribution of individual atoms to the London dispersion energy of a chemical system. This scheme, termed Atomic Decomposition of London Dispersion energy (ADLD), provides a new lens for understanding and quantifying the role of London dispersion forces across many research disciplines, such as molecular chemistry, biology and materials science.

The selected illustrative examples demonstrated that such quantification is achievable with “gold-standard” coupled cluster accuracy. Our results also reveal that London dispersion is highly sensitive to electronic structure effects such as spin state, charge, and resonance characteristics, which significantly modulate atomic contributions to dispersion energy, contrary to a widely held view. Case studies of benzene-lithium complexes highlight how ADLD can quantitatively capture charge-dependent variations in dispersion forces, revealing the advantages of wave function-based approaches for studying these effects over standard semiclassical schemes. Similarly, the analysis of *tert*-butyl-substituted cyclooctatetraene (COT) isomers elucidates the subtle interplay between σ–σ and σ–π dispersion interactions that govern the equilibrium in this system in various environments.

Our findings also clarify the origin of the recently proposed *gravitational-like* distance dependence of London dispersion energy. This behavior arises naturally from the dominance of *intramolecular* dispersion over *intermolecular* contributions and the energy scaling with system size. A generalized expression with broader validity was introduced.

Overall, ADLD emerges as a robust and versatile tool for quantifying and visualizing atomic dispersion contributions across diverse chemical scenarios. The insights afforded by this method have the potential to guide the design of molecules and materials, enabling precise manipulation of dispersion-driven properties and phenomena, such as conformational preferences, reaction selectivities and molecular recognition.

## Supplementary Material





## Notes

Additional computational details, supporting figures and tables, input file used for calculation of solid-state 1,4-di-Ad-COT, benchmarks, and Cartesian coordinates of all structures (PDF)
Transparent Peer Review report available (PDF)
