# Supplementary material for: A Quantum Chemical Method for Dissecting London Dispersion Energy into Atomic Building Blocks
Source: ACS Cent Sci. 2025 May 8;11(6):890–8. doi: 10.1021/acscentsci.5c00356 (PMC12203434; doi:10.1021/acscentsci.5c00356)
Supplement: Supplementary file 1 [file oc5c00356_si_001.pdf]

Electronic Supporting Information (ESI)

A Quantum Chemical Method for Dissecting London Dispersion Energy into Atomic Building Blocks

Gianluca Regni, Lorenzo Baldinelli, and Giovanni Bistoni*

Department of Chemistry, Biology and Biotechnology, University of Perugia Via Elce di Sotto, 8, 06123 Perugia (Italy). E-mail: giovanni.bistoni@unipg.it

Table of Contents

S1 Computational Details 2

S2 Calculation Results 2

S2.1 C_6H_6-Li and C_6H_6-Li^+ 2

S2.2 *t*Bu1,6 and *t*Bu1,4 5

S2.3 1,4-di-Ad-COT in solid-state 11

S2.4 Analysis of *Gravitational-like* Relationship of London Dispersion 26

S3 Input files 32

S4 Decomposition of Semiclassical Corrections 33

S5 Decomposition of Triples Corrections 34

S6 Study of Basis Set Convergence and the Influence of Population Schemes on Atomic Dispersion Contributions 34

S7 Geometric Coordinates 36

S1 Computational Details

All calculations were performed with a development version of ORCA quantum chemistry package based on version 6.1[1,2].

For C_6H_6-Li systems DFT calculations were carried out using B3LYP functional[3–6] and Ahlrichs' def2-QZVP basis set[7]. Grimme's D3[8] and D4[9,10] London dispersion corrections[11], in conjunction with Becke-Johnson damping function[12,13], were employed in the calculations. Couple-cluster[14] calculations were performed at the DLPNO-CCSD(T)[15–17] level in conjunction with LED[18–20]. The aug-cc-pCVTZ basis set[21] together with automatic constructed /C and /JK auxiliary basis sets[22] were used. Different thresholds from the default were set for SCF (ExtremeSCF) and PNO (TightPNO) calculations. All electron pairs were included in the coupled cluster treatment (TCutPairs = 0).

For gas-phase cyclooctatetraene-based systems, couple-cluster calculations were performed at the DLPNO-CCSD(T)[15–17] level in conjunction with LED[18–20]. The def2-QZVP basis set[7] together with matching /C and /JK auxiliary basis sets. All electron pairs were included in the coupled cluster treatment (TCutPairs = 0). The solid-state calculations were performed at DLPNO-CCSD level in conjunction with LED. The def2-TZVP(-f) basis set with matching /C and /J auxiliary basis set was used. RIJCOSX approximation and different thresholds for SCF calculation (VeryTightSCF), for estimated pair correlation energy (TCutPairs = 1e-5), and for orbital localization scheme (LocTol = 1.0e-6) were employed. Interactions between fragments, excluding the central monomer, are computed at HF level (See Section S3 for the input file used).

For calculation in the Section 2.3.3, DFT calculations were carried out using PBE0 functional[23] and cc-pVTZ basis set[21]. Grimme's D4 correction[9,10], in conjunction with Becke-Johnson damping function[12,13], were employed in the calculations. Couple-cluster calculations were performed at the DLPNO-CCSD(T) level in conjunction with LED. The cc-pVTZ basis set[21] together with matching /C and /JK auxiliary basis sets were used. Different thresholds from the default were set for PNO (TightPNO) calculations. Foster-Boys localization scheme was employed.

The tools for computing the ρ_{disp} and $\Delta\rho_{disp}$ are available online[24]. In the atomic decomposition analysis at the DLPNO–CCSD(T)/DLPNO-CCSD level, an orbital population cutoff of 0.1 electrons was applied. Specifically, any atomic charges smaller than 0.1 electrons were set to zero, and the remaining values were normalized. With this cutoff, upon Foster-Boys localization[25], the contribution of each electron is distributed only on a limited set of atoms. The dispersion (difference) density functions are in kcal mol^{-1} $Bohr^{-3}$ and they were evaluated on the ball-and-stick surfaces.

S2 Calculation Results

S2.1 C_6H_6-Li and C_6H_6-Li^+

Table S1. For C_6H_6-Li, Li dispersion contributions in kcal/mol at various *R* (Å), computed at different levels of theory. R is the distance between Li and the center of mass of benzene.

R	D3(BJ)	D4	DLPNO-CCSD(T) (Loewdin)	DLPNO-CCSD(T) (Mulliken)
1.9	-1.83	-0.53	-1.91	-1.91
2.1	-1.78	-0.54	-1.62	-1.62
2.3	-1.81	-0.87	-1.45	-1.45
2.5	-1.81	-1.10	-1.29	-1.29
2.689	-2.73	-3.70	-1.20	-1.20
2.9	-3.82	-3.48	-1.12	-1.12
3.1	-3.60	-3.13	-0.95	-0.95
3.3	-3.15	-2.75	-0.80	-0.80
3.5	-2.67	-2.36	-0.68	-0.68
3.7	-2.21	-1.98	-0.62	-0.62

Table S2. For C_6H_6-Li^+, Li^+ dispersion contributions in kcal/mol at various *R* (Å), computed at different levels of theory. R is the distance between Li and the center of mass of benzene.

R	D3(BJ)	D4	DLPNO-CCSD(T) (Loewdin)	DLPNO-CCSD(T) (Mulliken)
1.9	-1.83	-0.49	-0.38	-0.38
2.1	-1.78	-0.49	-0.25	-0.25
2.3	-1.81	-0.80	-0.15	-0.15
2.5	-1.81	-1.01	-0.09	-0.09
2.689	-2.73	-3.41	-0.06	-0.06
2.9	-3.82	-3.21	-0.03	-0.03
3.1	-3.60	-2.89	-0.02	-0.02
3.3	-3.15	-2.54	-0.01	-0.01
3.5	-2.67	-2.18	-0.01	-0.01
3.7	-2.21	-1.82	-0.02	-0.02

Table S3. Atomic dispersion contributions associated with the interaction between C_6H_6 and Li in kcal/mol at DLPNO-CCSD(T)/LED level.

	DLPNO-CCSD(T) (Loewdin)	DLPNO-CCSD(T) (Mulliken)
C	-0.245	-0.256
C	-0.247	-0.259
C	-0.246	-0.257
C	-0.246	-0.258
C	-0.245	-0.256
C	-0.246	-0.258
H	-0.023	-0.011
H	-0.024	-0.012
H	-0.023	-0.011
H	-0.024	-0.011
H	-0.023	-0.011
H	-0.024	-0.011
Li	-1.202	-1.202

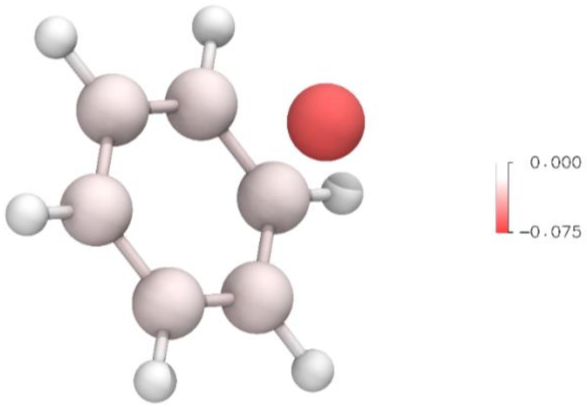

Figure S1. ρ_{disp} (α=0.5) of C_6H_6-Li at the DLPNO-CCSD(T)/LED level using Löwdin population analysis.

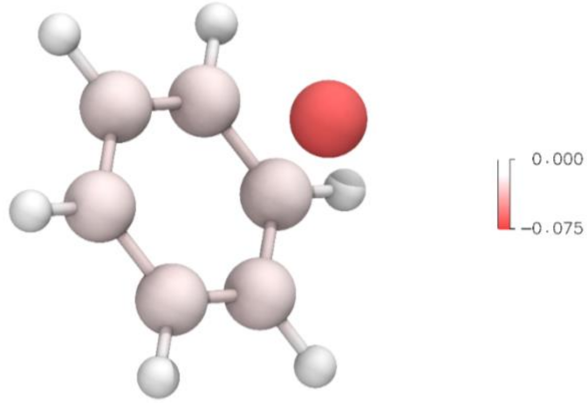

Figure S2. ρ_{disp} (α=0.5) of C_6H_6-Li at the DLPNO-CCSD(T)/LED level using Mulliken population analysis

Table S4. Atomic dispersion contributions associated with the interaction between C_6H_6 and Li^+ in kcal/mol at DLPNO-CCSD(T)/LED level.

	DLPNO-CCSD(T) (Loewdin)	DLPNO-CCSD(T) (Mulliken)
C	-0.015	-0.015
C	-0.015	-0.015
C	-0.015	-0.015
C	-0.015	-0.015
C	-0.014	-0.015
C	-0.015	-0.015
H	-0.001	0.000
H	-0.001	0.000
H	-0.001	0.000
H	-0.001	0.000
H	-0.001	0.000
H	-0.001	0.000
Li	-0.089	-0.089

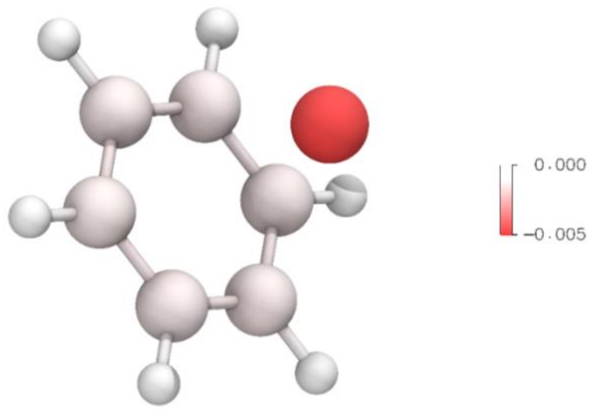

Figure S3. ρ_{disp} (α=0.5) of C_6H_6-Li^+ at the DLPNO-CCSD(T)/LED level using Löwdin population analysis.

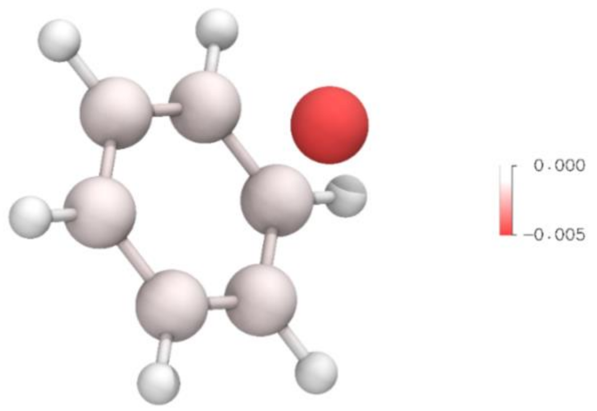

Figure S4. ρ_{disp} (α=0.5) of C_6H_6-Li^+ at the DLPNO-CCSD(T)/LED level using Mulliken population analysis.

Table S5. Single point energies of C_6H_6-Li, C_6H_6-Li^+ and their respective fragments and the interaction energy between benzene e lithium atom computed with a supramolecular approach in Hartree at different *R* (Å).

R	C_6H_6	Li	Li^+	C_6H_6-Li	C_6H_6-Li^+	E_{int} (C_6H_6-Li)	E_{int} (C_6H_6-Li^+)
1.9	-231.8231	-7.4746	-7.2769	-239.3014	-239.1608	-0.0038	-0.0608
2.1	-231.8231	-7.4746	-7.2769	-239.3038	-239.1579	-0.0062	-0.0579
2.3	-231.8231	-7.4746	-7.2769	-239.3041	-239.1527	-0.0065	-0.0527
2.5	-231.8231	-7.4746	-7.2769	-239.3036	-239.1465	-0.0059	-0.0466
2.689	-231.8231	-7.4746	-7.2769	-239.3028	-239.1408	-0.0052	-0.0408
2.9	-231.8231	-7.4746	-7.2769	-239.3021	-239.1348	-0.0044	-0.0348
3.1	-231.8231	-7.4746	-7.2769	-239.3015	-239.1298	-0.0038	-0.0298
3.3	-231.8231	-7.4746	-7.2769	-239.3010	-239.1255	-0.0034	-0.0255
3.5	-231.8231	-7.4746	-7.2769	-239.3006	-239.1218	-0.0029	-0.0219
3.7	-231.8231	-7.4746	-7.2769	-239.3002	-239.1187	-0.0026	-0.0188

R is the distance between Li and the center of mass of benzene.

S2.2 *t*Bu1,6 and *t*Bu1,4

Table S6. Atomic dispersion contributions of ***t*Bu1,6** in kcal/mol at DLPNO-CCSD(T)/LED level. Two tert-butyl groups and the central ring are defined as fragments.

	DLPNO-CCSD(T) (Loewdin)	DLPNO-CCSD(T) (Mulliken)
H	-0.15	-0.16
H	-0.30	-0.30
H	-0.29	-0.29
C	-1.78	-1.76
C	-1.78	-1.76
C	-2.12	-2.08
C	-5.51	-5.38
H	-0.38	-0.39
H	-0.18	-0.18
H	-0.25	-0.25
H	-0.28	-0.28
H	-0.22	-0.21
H	-0.16	-0.16
H	-0.22	-0.22
C	-1.80	-1.78
H	-0.38	-0.39
H	-0.15	-0.16
C	-1.75	-1.73
C	-2.12	-2.09
C	-5.51	-5.38
H	-0.28	-0.28
H	-0.24	-0.24
H	-0.30	-0.30
H	-0.18	-0.18
C	-3.98	-4.13
C	-1.69	-1.72
C	-0.32	-0.33
C	-1.66	-1.69
C	-1.65	-1.68
C	-0.32	-0.33
C	-1.69	-1.72
C	-3.98	-4.13
H	-0.32	-0.31
H	-0.32	-0.31
H	-0.04	-0.03
H	-0.04	-0.03
H	-0.34	-0.33

H	-0.33	-0.32
H	-0.29	-0.29
H	-0.15	-0.16

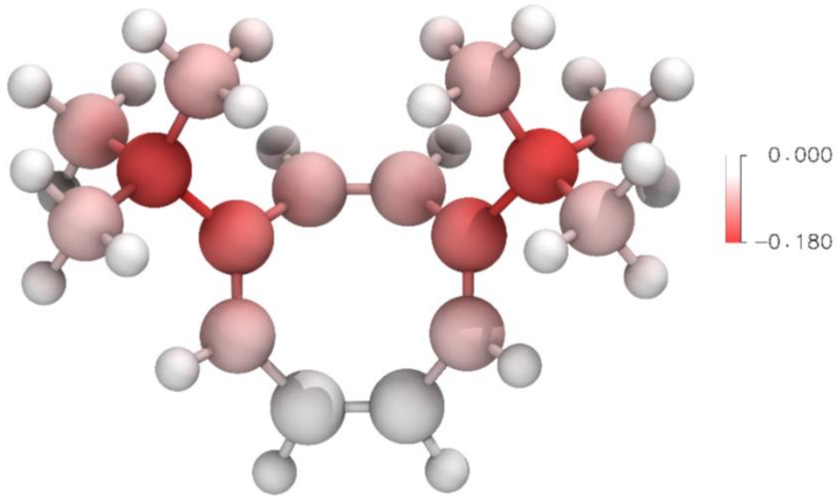

Figure S5. ρ_{disp} (α=0.3) of tBu1,6 at the DLPNO-CCSD(T)/LED level using Löwdin population analysis.

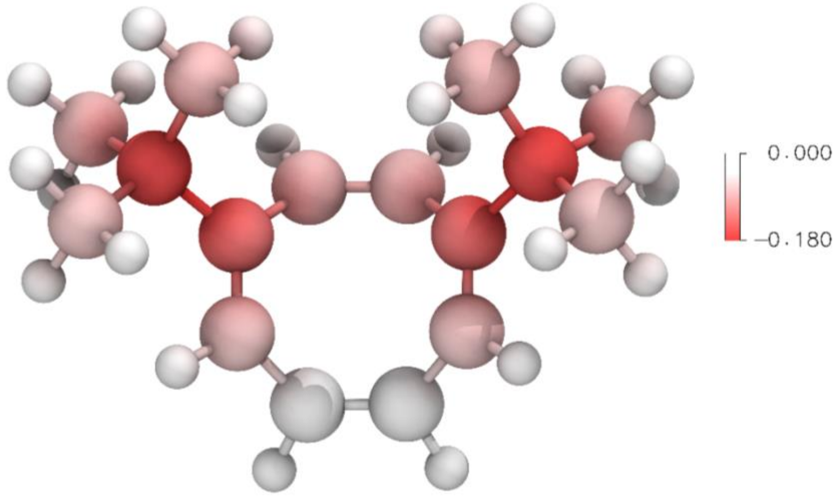

Figure S6. ρ_{disp} (α=0.3) of tBu1,6 at DLPNO-CCSD(T)/LED level using Mulliken population analysis.

Table S7. Atomic dispersion contributions of *t*Bu1,4 in kcal/mol at DLPNO-CCSD(T)/LED level. Two tert-butyl groups and the central ring are defined as fragments.

	DLPNO-CCSD(T) (Loewdin)	DLPNO-CCSD(T) (Mulliken)
H	-0.16	-0.16
H	-0.29	-0.29
H	-0.21	-0.21
C	-1.88	-1.85
C	-1.76	-1.75
C	-1.84	-1.82
C	-5.49	-5.34
H	-0.27	-0.27
H	-0.16	-0.16

H	-0.31	-0.31
H	-0.22	-0.21
H	-0.29	-0.29
H	-0.15	-0.16
H	-0.31	-0.32
C	-1.80	-1.78
H	-0.29	-0.29
H	-0.16	-0.16
C	-1.91	-1.89
C	-1.82	-1.81
C	-5.60	-5.45
H	-0.21	-0.21
H	-0.29	-0.29
H	-0.27	-0.27
H	-0.16	-0.16
C	-4.00	-4.17
C	-1.28	-1.31
C	-0.65	-0.66
C	-1.85	-1.89
C	-1.86	-1.89
C	-0.65	-0.66
C	-1.29	-1.32
C	-4.05	-4.23
H	-0.37	-0.35
H	-0.36	-0.35
H	-0.06	-0.06
H	-0.06	-0.06
H	-0.29	-0.28
H	-0.29	-0.28
H	-0.22	-0.22
H	-0.16	-0.16

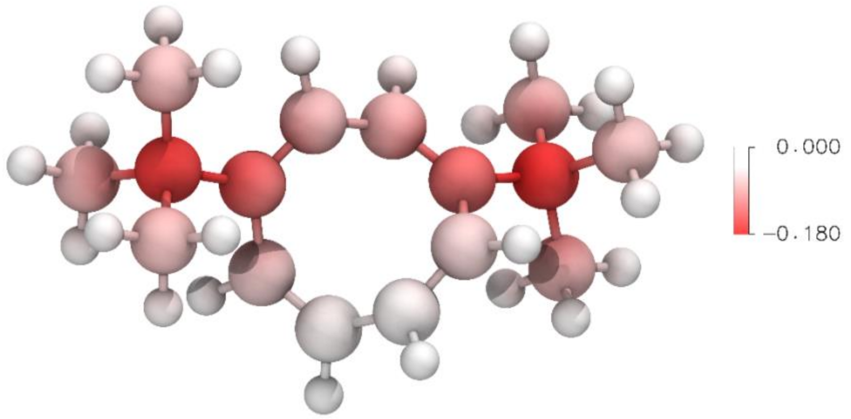

Figure S7. ρ_{disp} (α=0.3) of tBu1,4 at the DLPNO-CCSD(T)/LED level using Löwdin population analysis.

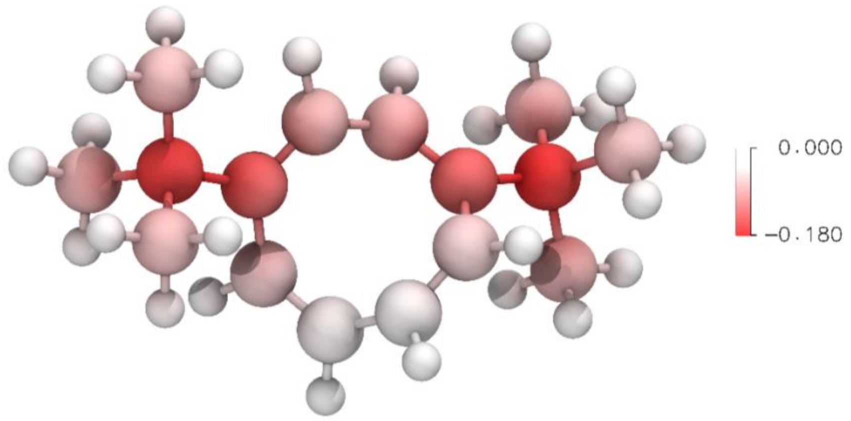

Figure S8. ρ_{disp} (α=0.3) of tBu1,4 at the DLPNO-CCSD(T)/LED level using Mulliken population analysis

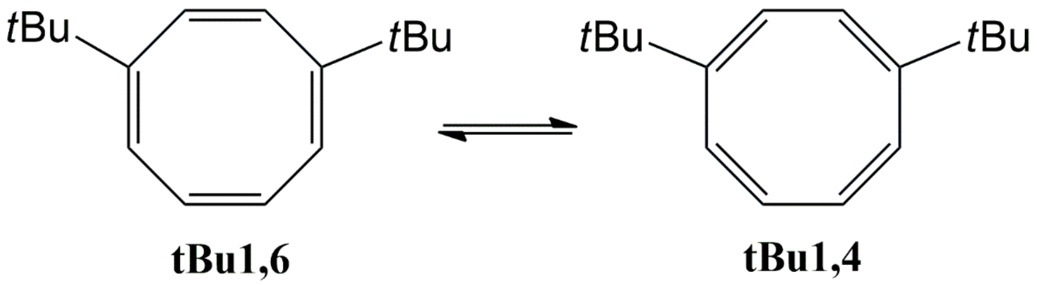

Figure S9. Equilibrium between 1,6-*t*Bu-cyclooctatetraene (tBu1,6) and 1,4-*t*Bu-cyclooctatetraene (tBu1,4).

The isomeric equilibrium between 1,6-di-tertbutyl-COT (***t*Bu1,6**) and 1,4-di-tertbutyl-COT (***t*Bu1,4**) in solution is governed by intramolecular London dispersion forces, making the sterically more crowded ***t*Bu1,6** more stable than the ***t*Bu1,4**, both in gas phase and in a wide range of solvents.[26] The main chemical mechanism behind this effect has already been investigated in previous works. Specifically, previous analysis suggested that the key dispersion interactions operating in this system can be categorized as σ-σ and σ-π dispersion interactions,[27] which are displayed in Figure S10.

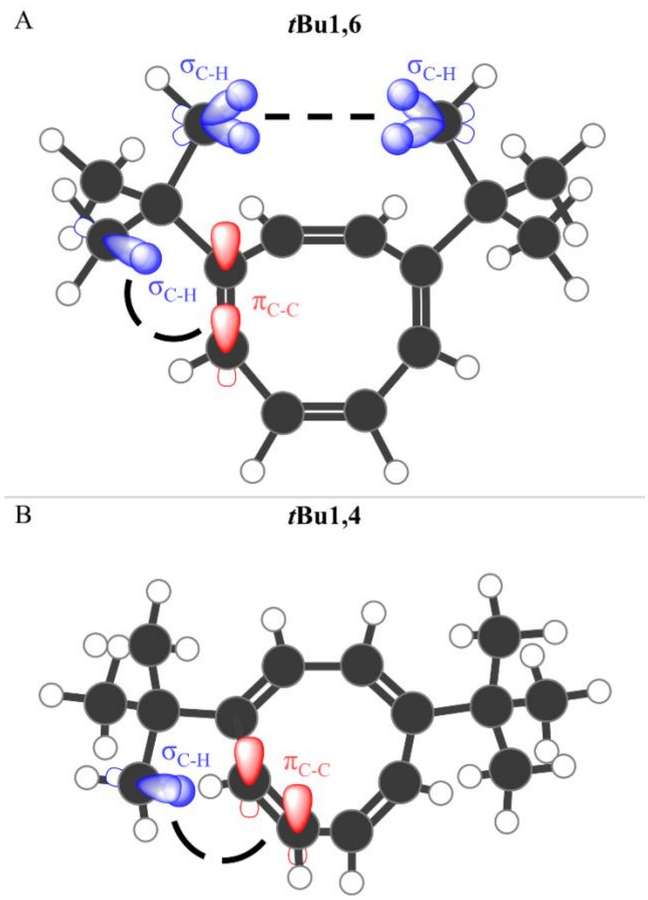

Figure S10. σ-σ and σ-π dispersion interactions in ***t*Bu1,6** (A) and ***t*Bu1,4** (B).

The greater stability of more crowded ***t*Bu1,6** isomer was attributed to the σ-σ CH-CH dispersion interactions between the tertbutyl groups in a series of recent works.[26–28] However, the ability to isolate London dispersion from other correlation effects within the ADLD(LED) now enables a deeper analysis.

A preliminary qualitative insight can be gained through visual inspection of ρ_{disp} for ***t*Bu1,6** (Figure S11A), ***t*Bu1,4** (Figure S11B) and the corresponding $\Delta\rho_{disp}$ (Figure S11C).

Analysis of ρ_{disp} reveals an intense red color for the quaternary carbons of tert-butyl groups and the tertiary carbons of the central ring to which they are bonded.

A **tBu1,6**

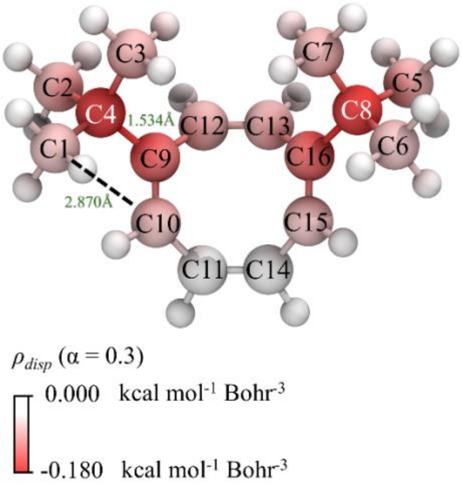

	ε_A^{disp} (kcal/mol)
C1	-1.76
C2	-1.76
C3	-2.08
C4	-5.38
C5	-1.78
C6	-1.73
C7	-2.09
C8	-5.38
C9	-4.13
C10	-1.72
C11	-0.33
C12	-1.69
C13	-1.68
C14	-0.33
C15	-1.72
C16	-4.13

B **tBu1,4**

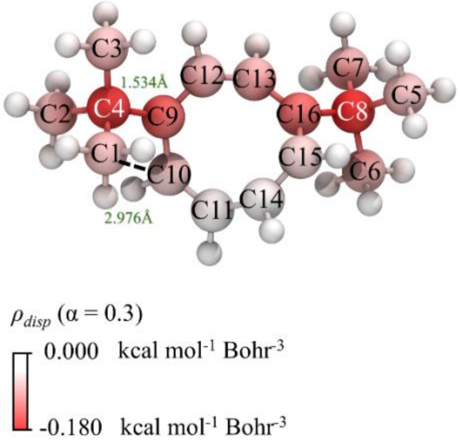

	ε_A^{disp} (kcal/mol)
C1	-1.89
C2	-1.78
C3	-1.81
C4	-5.45
C5	-1.75
C6	-1.85
C7	-1.82
C8	-5.34
C9	-4.23
C10	-1.32
C11	-0.66
C12	-1.89
C13	-1.89
C14	-0.66
C15	-1.31
C16	-4.17

C ***t*Bu1,6-*t*Bu1,4**

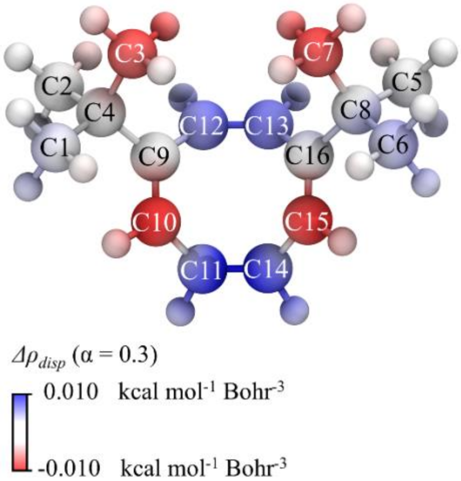

	$\Delta\varepsilon_A^{disp}$ (kcal/mol)
C1	0.09
C2	-0.02
C3	-0.27
C4	-0.04
C5	0.00
C6	0.16
C7	-0.28
C8	0.07
C9	0.04
C10	-0.41
C11	0.33
C12	020
C13	0.21
C14	0.33
C15	-0.40
C16	0.10

Figure S11. ADLD(LED) dispersion density function ρ_{disp} (α = 0.3) with the corresponding ε_A^{disp} for ***t*Bu1,6** (A) and ***t*Bu1,4** (B). (C) The corresponding dispersion density difference function $\Delta\rho_{disp}$ (***t*Bu1,6-*t*Bu1,4**) and $\Delta\varepsilon_A^{disp}$ (hydrogens are not shown for simplicity. The two tert-butyl groups and the central ring are defined as fragments in the LED calculations).

These results are consistent with the expected inverse dependence of dispersion by the interatomic distance and with the greater polarizability of carbon atoms with respect to that of hydrogen atoms.

Quantitatively, ADLD enables us to determine the dispersion contribution of each atom at the "gold standard" CCSD(T) level. The quaternary carbon atoms (C4 and C8) each contribute approximately 5 kcal/mol to the dispersion energy of both systems, while the tertiary carbon atoms (C9 and C16) contribute around 4 kcal/mol. These values are significantly larger compared to the differential stabilization of the two diastereomers, as measured experimentally or determined computationally. Specifically, the equilibrium enthalpy difference is about 0.3–0.6 kcal/mol, depending on the solvent.[26–28]

These results suggest that subtle variations in the contributions of individual atoms drive the isomeric preference. Therefore, this preference can be rationalized by closely analyzing the changes in atomic contributions between the two diastereomers.

S2.3 1,4-di-Ad-COT in solid-state

Table S8. Atomic dispersion contributions associated with the interaction between the central monomer and its neighboring monomers in a cluster model extracted from solid-state 1,4-di-Ad-COT at DLPNO-CCSD(T)/LED level. All energies are in kcal/mol.

	DLPNO-CCSD (Loewdin)	DLPNO-CCSD (Mulliken)
C	-0.34	-0.31
C	-0.54	-0.57
H	-0.14	-0.14
C	-0.55	-0.59
H	-0.15	-0.14
C	-0.34	-0.31
C	-0.59	-0.60
H	-0.13	-0.12
C	-0.77	-0.78
H	-0.22	-0.21
C	-0.78	-0.79
H	-0.23	-0.22
C	-0.59	-0.60
H	-0.13	-0.12
C	-0.22	-0.17
C	-0.52	-0.55
H	-0.13	-0.12
H	-0.16	-0.16
C	-0.66	-0.61
H	-0.25	-0.25
C	-0.58	-0.61
H	-0.16	-0.16
H	-0.14	-0.14
C	-0.48	-0.42
H	-0.15	-0.15
C	-0.49	-0.52
H	-0.14	-0.14
H	-0.14	-0.15
C	-0.53	-0.57
H	-0.16	-0.15
H	-0.12	-0.12
C	-0.67	-0.63
H	-0.24	-0.24

C	-0.77	-0.82
H	-0.19	-0.19
H	-0.22	-0.21
C	-0.72	-0.76
H	-0.21	-0.21
H	-0.18	-0.18
C	-0.22	-0.18
C	-0.47	-0.50
H	-0.15	-0.15
H	-0.11	-0.11
C	-0.46	-0.40
H	-0.13	-0.14
C	-0.59	-0.62
H	-0.15	-0.14
H	-0.16	-0.15
C	-0.66	-0.62
H	-0.25	-0.25
C	-0.56	-0.59
H	-0.16	-0.16
H	-0.15	-0.15
C	-0.54	-0.57
H	-0.15	-0.16
H	-0.15	-0.14
C	-0.67	-0.63
H	-0.25	-0.25
C	-0.74	-0.78
H	-0.22	-0.22
H	-0.20	-0.20
C	-0.76	-0.80
H	-0.19	-0.20
H	-0.21	-0.20
C	-0.01	-0.01
C	0.00	0.00
H	0.00	0.00
C	0.00	0.00
H	0.00	0.00
C	0.00	0.00
C	-0.18	-0.18
H	-0.01	-0.01
C	-0.35	-0.36
H	-0.10	-0.10
C	-0.38	-0.39
H	-0.12	-0.12
C	-0.19	-0.19
H	-0.01	-0.01
C	-0.02	-0.02
C	-0.16	-0.17
H	-0.08	-0.08

H	-0.02	-0.01
C	-0.15	-0.14
H	-0.06	-0.06
C	-0.02	-0.02
H	0.00	0.00
H	0.00	0.00
C	0.00	0.00
H	0.00	0.00
C	0.00	0.00
H	0.00	0.00
H	0.00	0.00
C	-0.01	-0.01
H	0.00	0.00
H	0.00	0.00
C	-0.03	-0.03
H	0.00	0.00
C	-0.20	-0.20
H	-0.09	-0.09
H	-0.02	-0.02
C	0.00	0.00
H	0.00	0.00
H	0.00	0.00
C	-0.03	-0.02
C	-0.20	-0.21
H	-0.03	-0.03
H	-0.09	-0.08
C	-0.20	-0.20
H	-0.09	-0.09
C	-0.03	-0.03
H	0.00	0.00
H	0.00	0.00
C	0.00	0.00
H	0.00	0.00
C	0.00	0.00
H	0.00	0.00
H	0.00	0.00
C	0.00	0.00
H	0.00	0.00
H	0.00	0.00
C	-0.02	-0.01
H	0.00	0.00
C	-0.13	-0.14
H	-0.06	-0.06
H	-0.01	-0.01
C	0.00	0.00
H	0.00	0.00
H	0.00	0.00
C	-0.12	-0.12

C	-0.12	-0.13
H	-0.01	-0.01
C	-0.12	-0.12
H	-0.05	-0.04
C	-0.05	-0.04
C	0.00	0.00
H	0.00	0.00
C	0.00	0.00
H	0.00	0.00
C	-0.04	-0.04
H	0.00	0.00
C	-0.11	-0.11
H	-0.04	-0.04
C	-0.06	-0.05
C	-0.01	-0.01
H	0.00	0.00
H	0.00	0.00
C	0.00	0.00
H	0.00	0.00
C	0.00	0.00
H	0.00	0.00
H	0.00	0.00
C	-0.05	-0.04
H	0.00	0.00
C	-0.02	-0.02
H	-0.01	-0.01
H	0.00	0.00
C	-0.31	-0.33
H	-0.14	-0.13
H	-0.04	-0.04
C	-0.24	-0.23
H	-0.09	-0.09
C	-0.03	-0.03
H	0.00	0.00
H	0.00	0.00
C	-0.30	-0.31
H	-0.13	-0.13
H	-0.05	-0.05
C	-0.05	-0.04
C	-0.01	-0.01
H	0.00	0.00
H	0.00	0.00
C	-0.02	-0.02
H	0.00	0.00
C	-0.13	-0.14
H	-0.01	-0.01
H	-0.05	-0.05
C	-0.21	-0.21

H	-0.09	-0.09
C	-0.30	-0.31
H	-0.13	-0.13
H	-0.05	-0.05
C	0.00	0.00
H	0.00	0.00
H	0.00	0.00
C	0.00	0.00
H	0.00	0.00
C	0.00	0.00
H	0.00	0.00
H	0.00	0.00
C	-0.03	-0.03
H	0.00	0.00
H	0.00	0.00
C	-0.13	-0.13
C	-0.30	-0.31
H	-0.10	-0.10
C	-0.32	-0.33
H	-0.10	-0.10
C	-0.14	-0.14
C	0.00	0.00
H	0.00	0.00
C	0.00	0.00
H	0.00	0.00
C	0.00	0.00
H	0.00	0.00
C	0.00	0.00
H	0.00	0.00
C	-0.03	-0.03
C	0.00	0.00
H	0.00	0.00
H	0.00	0.00
C	0.00	0.00
H	0.00	0.00
C	-0.03	-0.03
H	0.00	0.00
H	0.00	0.00
C	-0.21	-0.20
H	-0.10	-0.09
C	-0.23	-0.24
H	-0.10	-0.10
H	-0.03	-0.03
C	0.00	0.00
H	0.00	0.00
H	0.00	0.00
C	-0.02	-0.02
H	0.00	0.00

C	0.00	0.00
H	0.00	0.00
H	0.00	0.00
C	-0.14	-0.14
H	-0.06	-0.06
H	-0.02	-0.02
C	-0.03	-0.03
C	0.00	0.00
H	0.00	0.00
H	0.00	0.00
C	0.00	0.00
H	0.00	0.00
C	-0.02	-0.02
H	0.00	0.00
H	0.00	0.00
C	-0.18	-0.16
H	-0.07	-0.07
C	-0.21	-0.22
H	-0.02	-0.02
H	-0.10	-0.10
C	-0.02	-0.02
H	0.00	0.00
H	-0.01	-0.01
C	-0.03	-0.03
H	0.00	0.00
C	0.00	0.00
H	0.00	0.00
H	0.00	0.00
C	-0.22	-0.22
H	-0.10	-0.10
H	-0.03	-0.03
C	-0.05	-0.04
C	-0.10	-0.11
H	-0.04	-0.04
C	-0.10	-0.10
H	0.00	0.00
C	-0.10	-0.10
C	-0.10	-0.10
H	-0.04	-0.03
C	-0.04	-0.04
H	0.00	0.00
C	0.00	0.00
H	0.00	0.00
C	0.00	0.00
H	0.00	0.00
C	-0.03	-0.03
C	-0.01	-0.01
H	0.00	0.00

H	-0.01	-0.01
C	-0.02	-0.01
H	0.00	0.00
C	-0.13	-0.14
H	-0.06	-0.06
H	-0.01	-0.01
C	-0.13	-0.13
H	-0.05	-0.05
C	-0.23	-0.23
H	-0.03	-0.03
H	-0.11	-0.11
C	0.00	0.00
H	0.00	0.00
H	0.00	0.00
C	0.00	0.00
H	0.00	0.00
C	0.00	0.00
H	0.00	0.00
H	0.00	0.00
C	-0.02	-0.02
H	0.00	0.00
H	0.00	0.00
C	-0.06	-0.05
C	0.00	0.00
H	0.00	0.00
H	0.00	0.00
C	0.00	0.00
H	0.00	0.00
C	0.00	0.00
H	0.00	0.00
H	0.00	0.00
C	-0.02	-0.02
H	0.00	0.00
C	0.00	0.00
H	0.00	0.00
H	0.00	0.00
C	-0.31	-0.33
H	-0.06	-0.07
H	-0.12	-0.11
C	-0.27	-0.26
H	-0.13	-0.13
C	-0.04	-0.04
H	0.00	0.00
H	0.00	0.00
C	-0.16	-0.17
H	-0.06	-0.06
H	-0.03	-0.03
C	-0.02	-0.01

C	0.00	0.00
H	0.00	0.00
C	-0.01	-0.01
H	0.00	0.00
C	-0.02	-0.02
C	-0.02	-0.02
H	0.00	0.00
C	-0.04	-0.04
H	0.00	0.00
C	-0.31	-0.32
H	-0.10	-0.10
C	-0.27	-0.27
H	-0.08	-0.08
C	-0.02	-0.02
C	0.00	0.00
H	0.00	0.00
H	0.00	0.00
C	-0.02	-0.02
H	0.00	0.00
C	0.00	0.00
H	0.00	0.00
H	0.00	0.00
C	0.00	0.00
H	0.00	0.00
C	0.00	0.00
H	0.00	0.00
H	0.00	0.00
C	-0.18	-0.19
H	-0.02	-0.02
H	-0.08	-0.07
C	-0.24	-0.23
H	-0.11	-0.11
C	-0.17	-0.17
H	-0.06	-0.07
H	-0.03	-0.03
C	-0.03	-0.03
H	0.00	0.00
H	0.00	0.00
C	-0.04	-0.03
C	-0.24	-0.25
H	-0.12	-0.12
H	-0.03	-0.03
C	-0.12	-0.12
H	-0.04	-0.04
C	-0.13	-0.13
H	-0.01	-0.01
H	-0.06	-0.06
C	-0.02	-0.01

H	0.00	0.00
C	-0.02	-0.02
H	-0.01	-0.01
H	0.00	0.00
C	0.00	0.00
H	0.00	0.00
H	0.00	0.00
C	0.00	0.00
H	0.00	0.00
C	-0.01	-0.01
H	0.00	0.00
H	0.00	0.00
C	0.00	0.00
H	0.00	0.00
H	0.00	0.00
C	-0.01	-0.01
C	-0.01	-0.01
H	0.00	0.00
C	-0.01	-0.01
H	0.00	0.00
C	-0.02	-0.02
C	-0.30	-0.30
H	-0.09	-0.08
C	-0.34	-0.34
H	-0.11	-0.11
C	-0.05	-0.05
H	0.00	0.00
C	-0.02	-0.02
H	0.00	0.00
C	-0.05	-0.04
C	-0.31	-0.33
H	-0.05	-0.05
H	-0.14	-0.13
C	-0.20	-0.20
H	-0.08	-0.08
C	-0.13	-0.13
H	-0.06	-0.05
H	-0.01	-0.01
C	-0.02	-0.02
H	0.00	0.00
C	-0.01	-0.01
H	0.00	0.00
H	0.00	0.00
C	0.00	0.00
H	0.00	0.00
H	0.00	0.00
C	0.00	0.00
H	0.00	0.00

C	-0.03	-0.03
H	0.00	0.00
H	0.00	0.00
C	0.00	0.00
H	0.00	0.00
H	0.00	0.00
C	-0.02	-0.02
C	-0.01	-0.01
H	0.00	0.00
H	0.00	0.00
C	-0.05	-0.04
H	0.00	0.00
C	0.00	0.00
H	0.00	0.00
H	0.00	0.00
C	0.00	0.00
H	0.00	0.00
C	0.00	0.00
H	0.00	0.00
H	0.00	0.00
C	-0.18	-0.19
H	-0.09	-0.09
H	-0.02	-0.02
C	-0.21	-0.21
H	-0.09	-0.09
C	-0.30	-0.31
H	-0.13	-0.14
H	-0.05	-0.05
C	-0.03	-0.03
H	0.00	0.00
H	0.00	0.00
C	0.00	0.00
C	0.00	0.00
H	0.00	0.00
C	0.00	0.00
H	0.00	0.00
C	0.00	0.00
C	0.00	0.00
H	0.00	0.00
C	0.00	0.00
H	0.00	0.00
C	0.00	0.00
H	0.00	0.00
C	0.00	0.00
H	0.00	0.00
C	0.00	0.00
C	0.00	0.00
H	0.00	0.00

H	0.00	0.00
C	-0.03	-0.02
H	0.00	0.00
C	-0.04	-0.04
H	0.00	0.00
H	-0.02	-0.02
C	-0.04	-0.03
H	0.00	0.00
C	0.00	0.00
H	0.00	0.00
H	0.00	0.00
C	-0.02	-0.02
H	0.00	0.00
H	0.00	0.00
C	-0.12	-0.11
H	-0.04	-0.04
C	-0.20	-0.21
H	-0.02	-0.02
H	-0.10	-0.10
C	-0.23	-0.24
H	-0.02	-0.02
H	-0.11	-0.11
C	0.00	0.00
C	0.00	0.00
H	0.00	0.00
H	0.00	0.00
C	0.00	0.00
H	0.00	0.00
C	0.00	0.00
H	0.00	0.00
H	0.00	0.00
C	0.00	0.00
H	0.00	0.00
C	0.00	0.00
H	0.00	0.00
H	0.00	0.00
C	0.00	0.00
H	0.00	0.00
H	0.00	0.00
C	0.00	0.00
H	0.00	0.00
C	0.00	0.00
H	0.00	0.00
H	0.00	0.00
C	0.00	0.00
H	0.00	0.00
H	0.00	0.00
C	0.00	0.00

C	0.00	0.00
H	0.00	0.00
C	0.00	0.00
H	0.00	0.00
C	0.00	0.00
C	0.00	0.00
H	0.00	0.00
C	0.00	0.00
H	0.00	0.00
C	0.00	0.00
H	0.00	0.00
C	0.00	0.00
H	0.00	0.00
C	0.00	0.00
C	-0.03	-0.03
H	0.00	0.00
H	0.00	0.00
C	-0.23	-0.21
H	-0.11	-0.11
C	-0.23	-0.24
H	-0.04	-0.04
H	-0.09	-0.09
C	-0.03	-0.03
H	0.00	0.00
C	0.00	0.00
H	0.00	0.00
H	0.00	0.00
C	0.00	0.00
H	0.00	0.00
H	0.00	0.00
C	-0.02	-0.02
H	0.00	0.00
C	-0.14	-0.15
H	-0.02	-0.02
H	-0.06	-0.06
C	0.00	0.00
H	0.00	0.00
H	0.00	0.00
C	0.00	0.00
C	0.00	0.00
H	0.00	0.00
H	0.00	0.00
C	0.00	0.00
H	0.00	0.00
C	0.00	0.00
H	0.00	0.00
H	0.00	0.00
C	0.00	0.00

H	0.00	0.00
C	0.00	0.00
H	0.00	0.00
H	0.00	0.00
C	0.00	0.00
H	0.00	0.00
H	0.00	0.00
C	0.00	0.00
H	0.00	0.00
C	0.00	0.00
H	0.00	0.00
H	0.00	0.00
C	0.00	0.00
H	0.00	0.00
H	0.00	0.00
C	0.00	0.00
C	0.00	0.00
H	0.00	0.00
C	0.00	0.00
H	0.00	0.00
C	0.00	0.00
C	0.00	0.00
H	0.00	0.00
C	0.00	0.00
H	0.00	0.00
C	0.00	0.00
H	0.00	0.00
C	0.00	0.00
H	0.00	0.00
C	0.00	0.00
C	0.00	0.00
H	0.00	0.00
H	0.00	0.00
C	0.00	0.00
H	0.00	0.00
C	0.00	0.00
H	0.00	0.00
H	0.00	0.00
C	0.00	0.00
H	0.00	0.00
C	0.00	0.00
H	0.00	0.00
H	0.00	0.00
C	0.00	0.00
H	0.00	0.00
H	0.00	0.00
C	0.00	0.00
H	0.00	0.00

C	0.00	0.00
H	0.00	0.00
H	0.00	0.00
C	0.00	0.00
H	0.00	0.00
H	0.00	0.00
C	0.00	0.00
C	0.00	0.00
H	0.00	0.00
H	0.00	0.00
C	-0.03	-0.03
H	0.00	0.00
C	-0.24	-0.25
H	-0.10	-0.10
H	-0.04	-0.04
C	-0.21	-0.20
H	-0.09	-0.09
C	-0.02	-0.03
H	0.00	0.00
H	0.00	0.00
C	0.00	0.00
H	0.00	0.00
H	0.00	0.00
C	-0.02	-0.02
H	0.00	0.00
C	-0.01	-0.01
H	0.00	0.00
H	0.00	0.00
C	-0.14	-0.15
H	-0.01	-0.01
H	-0.07	-0.06
C	0.00	0.00
C	0.00	0.00
H	0.00	0.00
C	0.00	0.00
H	0.00	0.00
C	0.00	0.00
C	0.00	0.00
H	0.00	0.00
C	0.00	0.00
H	0.00	0.00
C	0.00	0.00
H	0.00	0.00
C	0.00	0.00
H	0.00	0.00
C	0.00	0.00
C	0.00	0.00
H	0.00	0.00

H	0.00	0.00
C	0.00	0.00
H	0.00	0.00
C	0.00	0.00
H	0.00	0.00
H	0.00	0.00
C	0.00	0.00
H	0.00	0.00
C	0.00	0.00
H	0.00	0.00
H	0.00	0.00
C	0.00	0.00
H	0.00	0.00
H	0.00	0.00
C	0.00	0.00
H	0.00	0.00
C	0.00	0.00
H	0.00	0.00
H	0.00	0.00
C	0.00	0.00
H	0.00	0.00
H	0.00	0.00
C	0.00	0.00
C	0.00	0.00
H	0.00	0.00
H	0.00	0.00
C	-0.04	-0.03
H	0.00	0.00
C	-0.04	-0.04
H	-0.02	-0.02
H	0.00	0.00
C	-0.02	-0.02
H	0.00	0.00
C	0.00	0.00
H	0.00	0.00
H	0.00	0.00
C	-0.02	-0.02
H	0.00	0.00
H	0.00	0.00
C	-0.12	-0.11
H	-0.03	-0.03
C	-0.25	-0.26
H	-0.03	-0.03
H	-0.13	-0.13
C	-0.17	-0.17
H	-0.02	-0.02
H	-0.08	-0.08

S2.4 Analysis of the *Gravitational-like* Relationship for the London Dispersion Energy

Table S9. Summary of data used to study the *gravitational-like* relationship of the London dispersion energy. *R* denotes the distance between the two dimer centers of masses.

Index	*Dimer*	*R* D4 (Å)	*M1* (amu)	*M1* (amu)	E_{int}^{disp} DLPNO-CCSD(T) (kcal/mol)	E_{tot}^{disp} D4 (kcal/mol)	$E_{monomer\ 1}^{disp}$ D4 (kcal/mol)	$E_{monomer\ 2}^{disp}$ D4 (kcal/mol)	E_{int}^{disp} D4 (kcal/mol)
1	$C_{10}H_{10}$-$C_{10}H_{10}$	7.378	130.9	130.9	-0.77	-27.30	-13.21	-13.21	-0.88
2	$C_{14}H_{14}$-$C_{14}H_{14}$	8.150	182.3	182.3	-0.99	-44.46	-21.59	-21.59	-1.28
3	$C_{20}H_{20}$-$C_{20}H_{20}$	8.472	260.4	260.4	-1.35	-81.12	-39.59	-39.59	-1.94
4	C_7H_{16}-C_7H_{16}	4.944	100.2	100.2	-2.12	-19.28	-8.43	-8.43	-2.42
5	He-He	2.798	4.0	4.0	-0.02	-0.02	0.00	0.00	-0.02
6	$C_{20}H_{20}$-CH_4	6.123	260.4	16.0	-0.54	-40.99	-39.59	-0.44	-0.96
7	$C_{20}H_{20}$-C_4H_{10}	6.028	260.4	58.1	-3.95	-47.31	-39.59	-4.40	-3.31
8	$C_{20}H_{20}$-C_5H_{12}	6.289	260.4	72.1	-3.98	-49.53	-39.59	-6.53	-3.41
9	$C_{20}H_{20}$-C_8H_{10}	5.468	260.4	106.2	-6.61	-54.44	-39.59	-9.15	-5.70
10	Buckminsterfullerene-CH_4	6.840	720.7	16.0	-	-125.48	-122.93	-0.44	-123.37

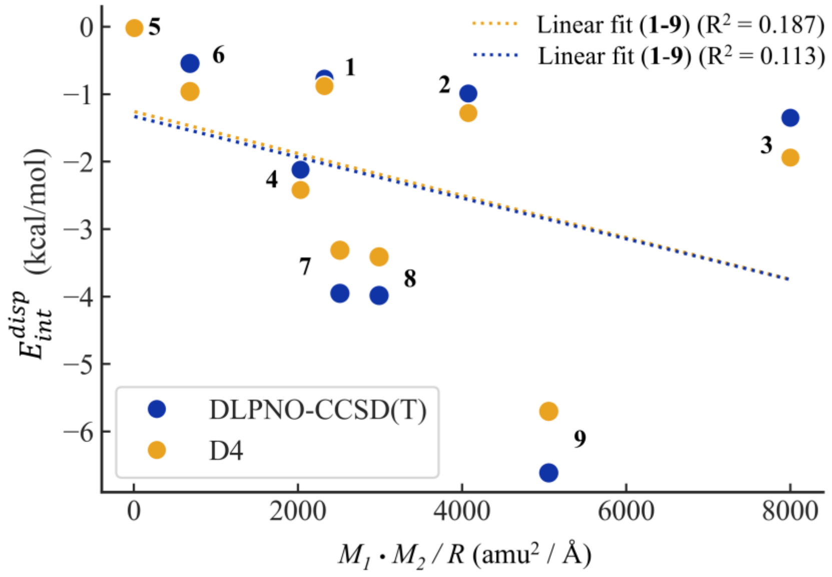

Figure S12. Dependence of dispersion interaction energy (E_{int}^{disp}) on ($M_1 \cdot M_2 / R$), where M_1 and M_2 are the molecular masses of the dimer and R is the distance between the respective centers of mass for **1-9** set of dimers. E_{int}^{disp} was computed at the DFT-D4 (yellow) and DLPNO-CCSD(T)/LED (blue) levels.

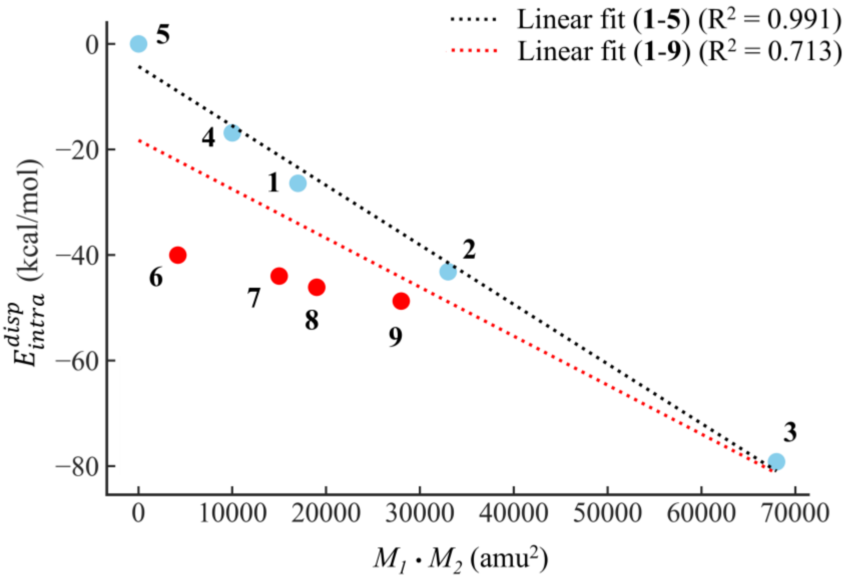

Figure S13. Dependence of intramolecular dispersion energy (E_{intra}^{disp}) on ($M_1 \cdot M_2$), where M_1 and M_2 are the molecular masses of the dimer, for **1-5** (blue) and **1-9** (red) set of dimers. E_{intra}^{disp} was computed at the DFT-D4 level.

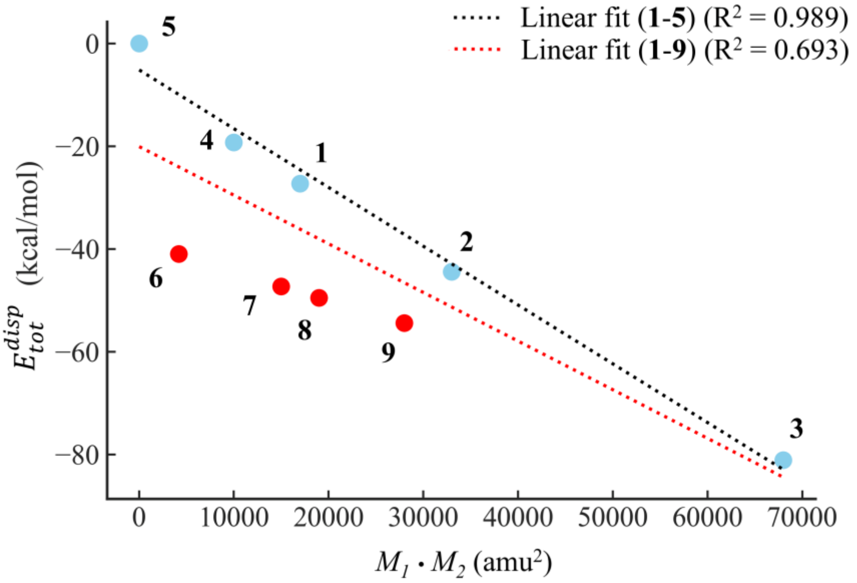

Figure S14. Dependence of total dispersion energy (E_{tot}^{disp}) on ($M_1 \cdot M_2$), where M_1 and M_2 are the molecular masses of the dimer, for **1-5** (blue) and **1-9** (red) set of dimers. E_{tot}^{disp} was computed at the DFT-D4 level.

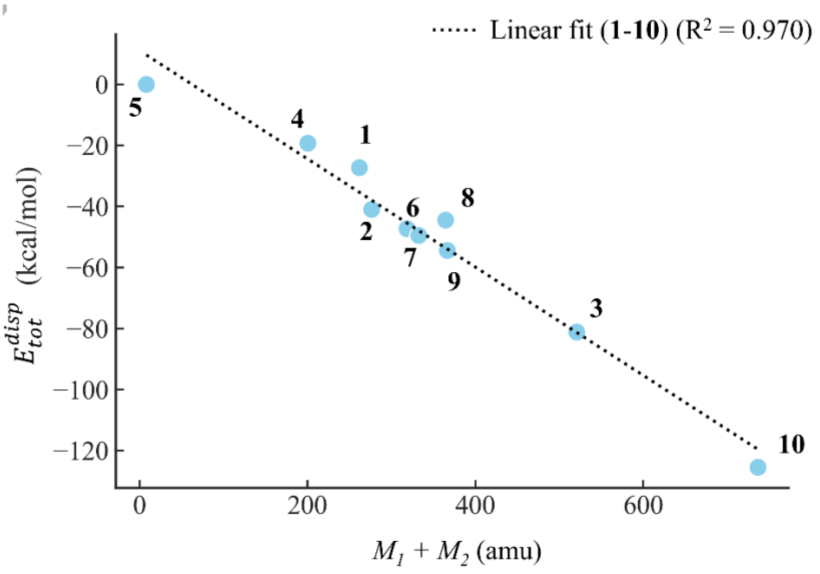

Figure S15. Dependence of total dispersion energy (E_{tot}^{disp}) on (M_1+M_2), where M_1 and M_2 are the molecular masses of the dimer, for **1-10** set of dimers. E_{tot}^{disp} was computed at the DFT-D4 level.

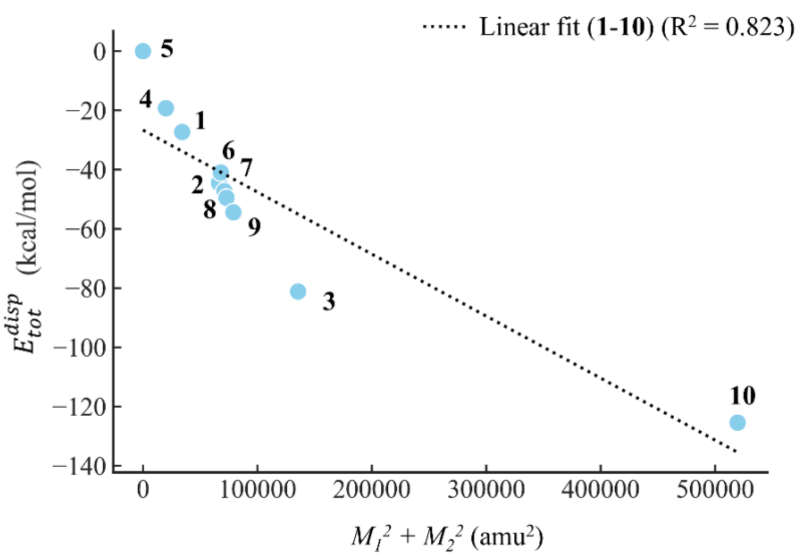

Figure S16. Dependence of total dispersion energy (E_{tot}^{disp}) on (M_1^2+M_2^2), where M_1 and M_2 are the molecular masses of the dimer, for ***1-10*** set of dimers. E_{tot}^{disp} was computed at the DFT-D4 level.

Dodecahedrane Dimer

Table S10. Atomic dispersion interaction of dodecahedrane dimer in kcal/mol at different levels of theory.

	E_{disp}^{int} DFT-D4	E_{disp}^{int} DLPNO-CCSD(T) (Loewdin)	E_{disp}^{int} DLPNO-CCSD(T) (Mulliken)
C	-0.01	0.00	0.00
C	-0.03	0.00	0.00
H	0.00	0.00	0.00
H	-0.01	0.00	0.00
C	-0.08	-0.05	-0.05
C	-0.03	0.00	0.00

H	-0.04	-0.02	-0.02
H	-0.01	0.00	0.00
C	-0.03	0.00	0.00
C	-0.03	0.00	0.00
H	-0.01	0.00	0.00
H	-0.01	0.00	0.00
C	-0.01	0.00	0.00
C	0.00	0.00	0.00
H	0.00	0.00	0.00
H	0.00	0.00	0.00
C	-0.03	0.00	0.00
H	-0.01	0.00	0.00
C	-0.01	0.00	0.00
H	0.00	0.00	0.00
C	-0.01	0.00	0.00
H	0.00	0.00	0.00
C	-0.08	-0.05	-0.05
H	-0.04	-0.02	-0.02
C	-0.08	-0.05	-0.05
H	-0.04	-0.02	-0.02
C	-0.01	0.00	0.00
H	0.00	0.00	0.00
C	-0.01	0.00	0.00
H	0.00	0.00	0.00
C	-0.03	0.00	0.00
H	-0.01	0.00	0.00
C	-0.01	0.00	0.00
H	0.00	0.00	0.00
C	-0.01	0.00	0.00
H	0.00	0.00	0.00
C	-0.01	0.00	0.00
H	0.00	0.00	0.00
C	-0.17	-0.27	-0.29
H	-0.13	-0.18	-0.16
H	-0.13	-0.18	-0.16
C	-0.17	-0.27	-0.29
C	-0.08	-0.05	-0.05
C	-0.08	-0.05	-0.05
C	-0.08	-0.05	-0.05
C	-0.03	0.00	0.00
H	-0.04	-0.02	-0.02
C	-0.03	0.00	0.00
C	-0.03	0.00	0.00
C	-0.03	0.00	0.00
H	-0.04	-0.02	-0.02
C	-0.03	0.00	0.00
H	-0.04	-0.02	-0.02
C	-0.03	0.00	0.00

H	-0.01	0.00	0.00
C	-0.01	0.00	0.00
H	-0.01	0.00	0.00
C	-0.01	0.00	0.00
H	-0.01	0.00	0.00
C	-0.01	0.00	0.00
H	-0.01	0.00	0.00
C	-0.01	0.00	0.00
C	-0.01	0.00	0.00
H	-0.01	0.00	0.00
H	-0.01	0.00	0.00
C	-0.01	0.00	0.00
C	-0.01	0.00	0.00
H	0.00	0.00	0.00
C	-0.01	0.00	0.00
H	0.00	0.00	0.00
H	0.00	0.00	0.00
C	-0.01	0.00	0.00
H	0.00	0.00	0.00
H	0.00	0.00	0.00
H	0.00	0.00	0.00
C	0.00	0.00	0.00
H	0.00	0.00	0.00
H	0.00	0.00	0.00
H	0.00	0.00	0.00
H	0.00	0.00	0.00

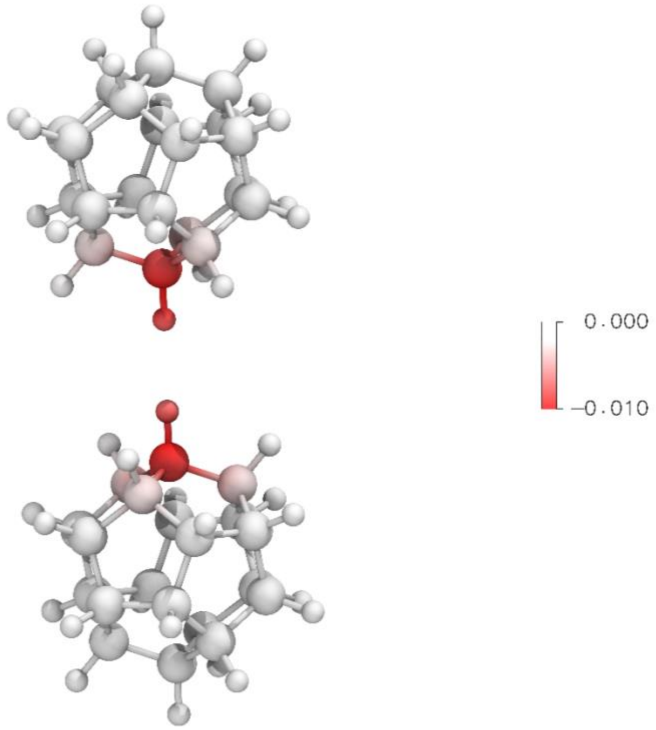

Figure S17. ρ_{disp} (α=0.3) of dodecahedrane dimer at the DLPNO-CCSD(T) level using Löwdin population analysis

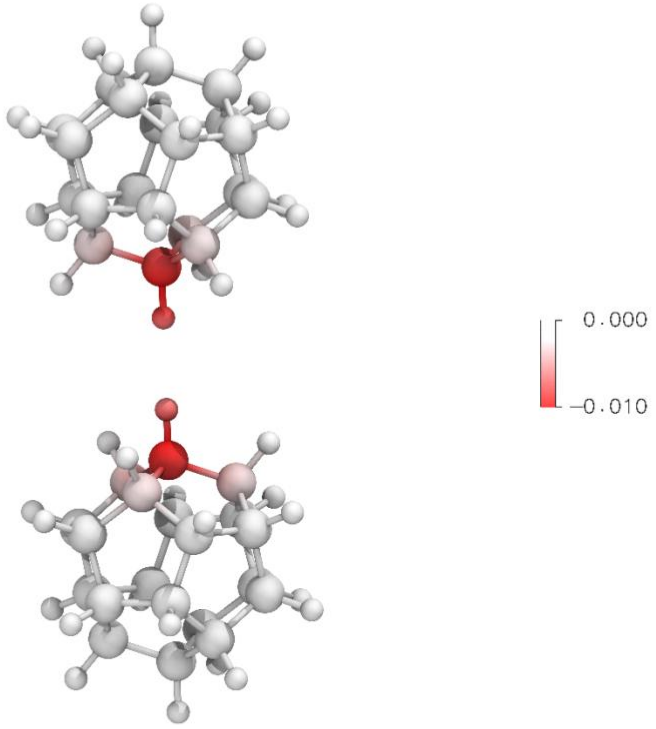

Figure S18. ρ_{disp} (α=0.3) of dodecahedrane dimer at the DLPNO-CCSD(T) level using Mulliken population analysis

S3 Input files

Listing S1. Sample input file to perform a ADLD(D4) calculation with ORCA (using a development version of ORCA based on ORCA 6.1).

```
! B3LYP D4 def2-QZVP ADLD

* xyzfile 0 2 BenzLi.xyz
```

Listing S2. Sample input file to perform a DLPNO-CCSD(T) calculation in conjunction with ADLD(LED) scheme with ORCA (using a development version of ORCA based on ORCA 6.1).

```
! DLPNO-CCSD(T) def2-QZVP def2-QZVPP/C def2/JK LED ADLD

%mdci
        AD_Mulliken  true
        AD_Loewdin  true
        TCutPairs                     0
end

*xyz 0 1
   H(1)        0.75870        0.59326        4.31290
   H(1)        0.37765        1.12381        1.36809
   H(1)       -1.24727       -2.03033        2.62851
   C(1)        1.37950       -0.01766        3.64861
[...]
*
```

Listing S3. Input file used for calculation of solid-state 1,4-di-Ad-COT with OR, (using a development version of ORCA based on ORCA 6.1).

```
! DLPNO-CCSD RIJCOSX def2-TZVP(-f) def2/J def2-tzvp/C verytightscf LED ADLD

%scf maxiter 999 end
%mdci TCutPairs 1e-5 printlevel 3
      LocMet FB LocTol 1.0e-6 LocRandom 1
      HFFragInter {1 1} {2 2} {3 3} {4 4} {5 5} {6 6} {7 7} {8 8} {9 9} {10 10} {11 11}
                  {2 3} {2 4} {2 5} {2 6} {2 7} {2 8} {2 9} {2 10} {2 11}
                  {3 4} {3 5} {3 6} {3 6} {3 8} {3 9} {3 10} {3 11}
                  {4 5} {4 6} {4 7} {4 8} {4 9} {4 10} {4 11}
                  {5 6} {5 7} {5 8} {5 9} {5 10} {5 11}
                  {6 7} {6 8} {6 9} {6 10} {6 11}
                  {7 8} {7 9} {7 10} {7 11}
                  {8 9} {8 10} {8 11}
                  {9 10} {9 11}
                  {10 11}
      AD_Mulliken true
      AD_Loewdin  true
end

*xyz 0 1
C(1)       3.959814    2.561396    5.671998
C(1)       5.124688    2.711507    6.307297
H(1)       5.910980    2.624495    5.781307
[...]
*
```

S4 Decomposition of Semiclassical Corrections

Mean-field methods, such as DFT and semiempirical approaches, generally fail to accurately describe long-range correlation effects and thus cannot account for London Dispersion Energy[29]. To address this challenge, semiclassical dispersion corrections, which add a corrective term to the total energy, such as DFT-D, are often employed[30]. They can be typically written in terms of atoms-pairwise contributions ε_{AB}^{SC}:

$$E_{disp}^{SC}(AB) = \sum_{A>B} \varepsilon_{AB}^{SC} = \frac{1}{2}\sum_{A}\sum_{B\neq A} \varepsilon_{AB}^{SC} = \sum_{A} \varepsilon_{A}^{SC}(AB),$$

where A and B denote the atoms pair label. In some cases, dispersion corrections include a many-body term for effects such as three-body interactions. In these instances, the scheme can be easily generalized, and a corresponding expression for the decomposition of three-body effects is defined as

$$E_{disp}^{SC}(ABC) = \sum_{A>B>C} \varepsilon_{ABC}^{SC} = \frac{1}{6}\sum_{A}\sum_{B\neq A\neq C}\sum_{C\neq A\neq B} \varepsilon_{ABC}^{SC}$$

$$= \sum_{A} \varepsilon_A^{SC}(ABC).$$

Importantly, semiclassical dispersion analysis does not require electronic structure calculations, as it relies solely on the contributions from the dispersion corrections. This approach significantly accelerates the procedure, enabling the study of systems with several thousand atoms.

S5 Decomposition of Triples Corrections

A possible way of decomposing triples corrections into atomic contributions $\varepsilon_A^{disp,triples}$ is by exploiting the ratio between correlation and dispersion energies at the CCSD level. This ratio can be computed from different contributions:

- electron pair-wise contributions;
- individual electron contributions;
- atomic contributions.

Since each of these is arbitrary and the influence of the triples on the atomic dispersion contributions is minimal, the last one was chosen for simplicity in the present case:

$$\varepsilon_A^{disp,triples} = \gamma_A \varepsilon_A^{C,triples}$$

where γ_A is the ratio between the correlation and dispersion energies of atom A at the CCSD level; $\varepsilon_A^{C,triples}$ is the correlation energy contribution of atom A from the triples corrections, obtained partitioning in equal part the correlation contributions of each electron of the triples ε_{ijk}^{C} and using a charge partitioning scheme ω_{Ai} to map each contribution onto the atoms:

$$\varepsilon_A^{C,triples} = \sum_{i} \sum_{j \neq i \neq k} \sum_{k \neq i \neq j} \frac{1}{6} \varepsilon_{ijk}^{C} \omega_{Ai} = \sum_{i} \varepsilon_i^{C,triples} \omega_{Ai}.$$

S6 Study of Basis Set Convergence and the Influence of Population Schemes on Atomic Dispersion Contributions

Table S11. Basis set convergence of the atomic dispersion contributions at the ADLD(LED) level using Mulliken and Löwdin population schemes.

	cc-PVDZ Mulliken	cc-PVTZ Mulliken	cc-PVQZ Mulliken	cc-PVDZ Löwdin	cc-PVTZ Löwdin	cc-PVQZ Löwdin
C	-0.004	-0.006	-0.006	-0.004	-0.006	-0.006
C	-0.004	-0.006	-0.006	-0.004	-0.006	-0.006
C	-0.004	-0.006	-0.006	-0.004	-0.006	-0.006
C	-0.004	-0.006	-0.006	-0.004	-0.006	-0.006
C	-0.004	-0.006	-0.006	-0.004	-0.006	-0.006
C	-0.004	-0.006	-0.006	-0.004	-0.006	-0.006
H	0.000	-0.001	-0.001	0.000	-0.001	-0.001
H	0.000	-0.001	-0.001	0.000	-0.001	-0.001
H	0.000	-0.001	-0.001	0.000	-0.001	-0.001
H	0.000	-0.001	-0.001	0.000	-0.001	-0.001
H	0.000	-0.001	-0.001	0.000	-0.001	-0.001
H	0.000	-0.001	-0.001	0.000	-0.001	-0.001
Li	-0.024	-0.040	-0.040	-0.024	-0.040	-0.040

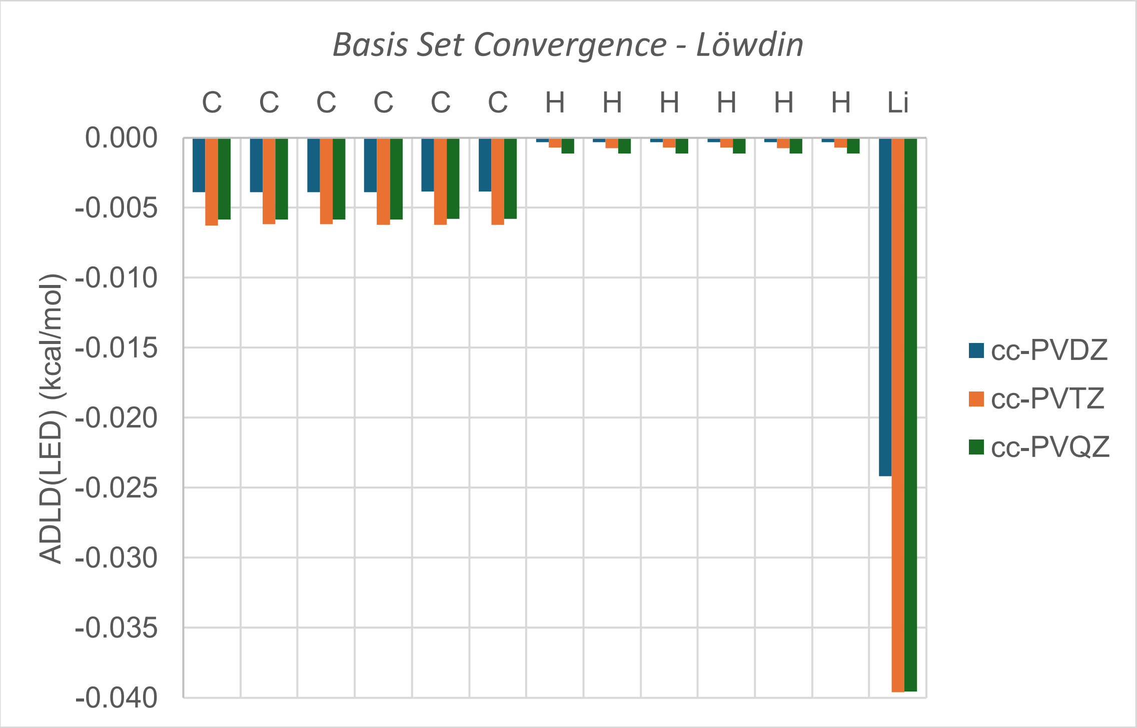

Figure S19. Basis set convergence histogram of atomic dispersion contributions at LED level using Löwdin population analysis.

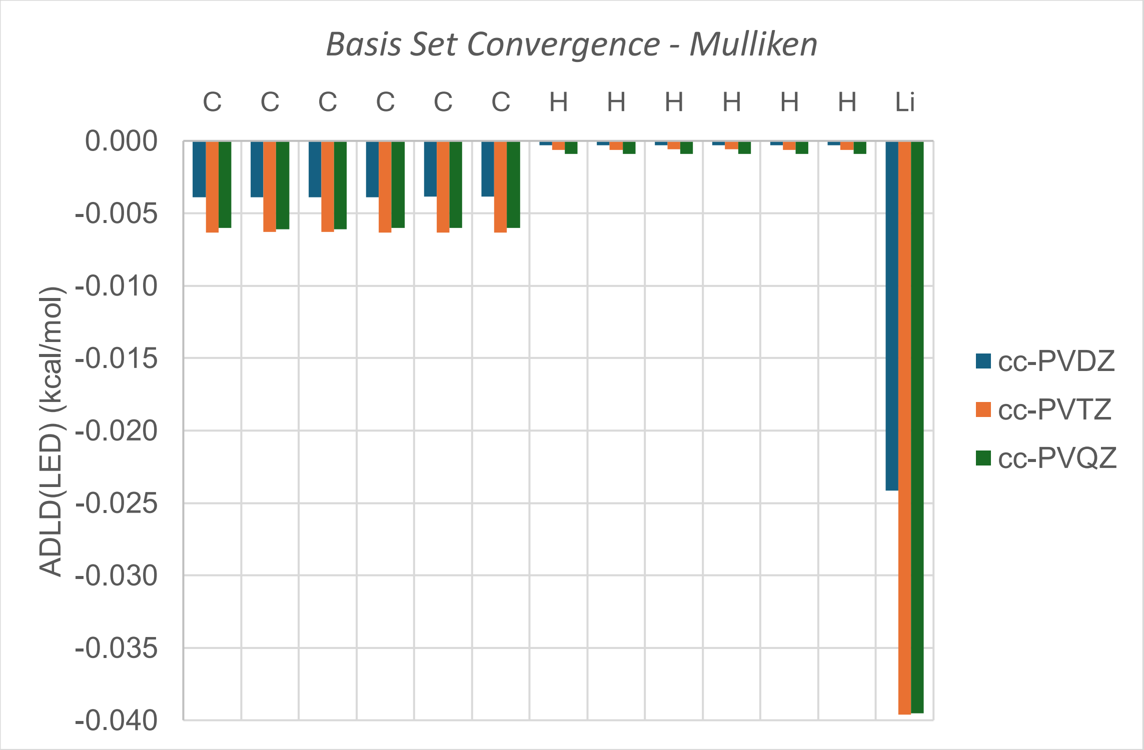

Figure S20. Basis set convergence histogram of atomic dispersion contributions at LED level using Mulliken population analysis.

.

S7 Geometric Coordinates

Table S12. Geometric coordinates of **C_6H_6-Li** optimized at B3LYP-D4/def2-TZVP level.

Atom	X	Y	Z
C	1.31455	-0.4565	0.00055
C	1.05272	0.91022	0.00303
C	-0.2619	1.3667	0.00264
C	-1.31455	0.45648	-0.00046
C	-1.05271	-0.91024	-0.0032
C	0.2619	-1.36671	-0.00264
H	2.33657	-0.81161	-0.00589
H	1.87126	1.61775	-0.00183
H	-1.87122	-1.61775	-0.0124
H	0.46562	-2.42928	-0.01146
H	-0.46562	2.4293	-0.00253
H	-2.33652	0.81168	-0.00783
Li	0	0	2.68969

For distance dependence calculations the molecular geometry was not optimized for each distance. The Li atom was displaced from the optimized equilibrium geometry of the neutral system by a fixed amount to avoid geometry-related effects.

Table S13. Geometric coordinates of ***t*Bu1,6** taken from ref[26].

Atom	X	Y	Z
H	0.7587	0.59326	4.3129
H	0.37765	1.12381	1.36809
H	-1.24727	-2.03033	2.62851
C	1.3795	-0.01766	3.64861
C	-0.49984	-1.61654	3.31179
C	-0.3019	0.4231	1.86419
C	0.49042	-0.67877	2.59013
H	-0.96352	0.00038	1.10193
H	-0.91479	0.98294	2.57999
H	1.89533	-0.75748	4.27003
H	2.12889	0.63705	3.19106
H	0.02953	-2.44783	3.78979
H	-1.03839	-1.06007	4.08746
H	1.19615	-1.5865	-4.19073
C	0.53267	-0.85338	-3.71945
H	-0.57838	0.28611	-1.44363
H	1.96607	1.46864	-3.84288
C	2.38919	0.71315	-3.17201
C	0.26134	0.83379	-1.88225
C	1.25424	-0.12111	-2.56883
H	2.96093	1.23376	-2.39634
H	3.07904	0.09985	-3.76157
H	0.75515	1.39132	-1.07961
H	-0.13752	1.55168	-2.6082

C	1.29668	-1.47819	1.55901
C	2.60596	-1.73656	1.70467
C	3.44116	-2.56932	0.83711
C	0.51536	-2.07791	0.45532
C	0.70995	-1.93633	-0.85867
C	3.63613	-2.42798	-0.48091
C	3.05913	-1.40757	-1.35817
C	1.75573	-1.14407	-1.54214
H	-0.34394	-2.67571	0.76228
H	-0.00644	-2.43034	-1.51647
H	4.04144	-3.31996	1.35427
H	4.38201	-3.07296	-0.94873
H	3.11557	-1.36984	2.59445
H	3.79072	-0.87965	-1.96808
H	-0.36747	-1.37216	-3.37692
H	0.22282	-0.12964	-4.482

Table S14. Geometric coordinates of ***t*Bu1,4** taken from ref[26].

Atom	X	Y	Z
H	-4.44962	-1.67822	-0.96457
H	-2.24388	-0.84572	-2.94879
H	-1.38609	-3.87694	-0.84959
C	-3.56454	-1.18563	-0.54552
C	-2.31231	-3.36314	-0.57003
C	-2.26666	-1.86958	-2.56069
C	-2.28267	-1.89108	-1.02903
H	-1.40871	-2.41631	-2.96689
H	-3.17462	-2.35157	-2.93904
H	-3.6429	-1.20824	0.54574
H	-3.56817	-0.13642	-0.8608
H	-2.44489	-3.45477	0.51194
H	-3.1508	-3.88375	-1.04659
H	1.87551	2.5176	2.30612
C	2.79871	3.06048	-0.2455
H	3.76193	-0.19138	0.46046
H	3.78532	3.4761	-0.01072
C	2.68138	1.86326	1.95986
C	3.8217	0.80593	0.01187
C	2.63768	1.68344	0.43006
H	2.71385	2.97235	-1.33409
H	2.05144	3.77986	0.10192
H	3.88342	0.69629	-1.07631
H	4.75411	1.27063	0.35031
C	-1.07133	-1.17681	-0.41616
C	-1.0585	-1.10571	1.06004

C	-0.99506	0.01317	1.79011
C	-0.06516	-0.68384	-1.15411
C	1.17179	-0.0595	-0.6671
C	-0.89115	1.37506	1.25782
C	0.09733	1.83516	0.48314
C	1.28458	1.06969	0.0486
H	2.08105	-0.54066	-1.02576
H	-0.09083	-0.83206	-2.23312
H	-1.65625	2.07949	1.5894
H	-1.09945	-0.07195	2.87335
H	0.0757	2.89108	0.21392
H	-1.19815	-2.04651	1.59225
H	2.56947	0.897	2.46393
H	3.63777	2.30518	2.26257

Table S15. Geometric coordinates of solid-state **1,4-di-Ad-COT** taken from ref[26].

Atom	X	Y	Z
C	3.959814	2.561396	5.671998
C	5.124688	2.711507	6.307297
H	5.91098	2.624495	5.781307
C	5.31875	3.001413	7.75616
H	5.764958	2.33804	8.269299
C	4.922108	4.116628	8.413564
C	4.187615	5.217888	7.728235
H	4.592983	6.076768	7.705738
C	2.9965	5.073177	7.144286
H	2.59824	5.854076	6.778112
C	2.24056	3.812194	7.014673
H	1.37463	3.783577	7.404357
C	2.667365	2.709888	6.398299
H	2.09633	1.951116	6.424481
C	3.855528	2.18951	4.180727
C	3.206858	3.36561	3.44357
H	2.312363	3.542338	3.829289
H	3.759862	4.17721	3.568431
C	3.066693	3.071653	1.950331
H	2.641663	3.853971	1.494983
C	4.418385	2.80199	1.331483
H	4.99231	3.603436	1.423104
H	4.310148	2.608715	0.366596
C	5.082741	1.613149	2.021845
H	5.984031	1.450291	1.620378
C	5.232054	1.929266	3.531282
H	5.671955	1.167507	3.985505
H	5.806469	2.727454	3.645513
C	2.98449	0.916534	3.972879

H	3.414105	0.144085	4.418806
H	2.094116	1.053032	4.383598
C	2.820046	0.619609	2.471962
H	2.244245	-0.18911	2.351826
C	2.172758	1.82021	1.768524
H	1.276608	1.990313	2.153342
H	2.063503	1.624174	0.804312
C	4.205273	0.362816	1.851089
H	4.633831	-0.41062	2.296313
H	4.107182	0.151182	0.888958
C	5.254459	4.351654	9.886415
C	3.942843	4.629858	10.62636
H	3.33715	3.853519	10.52374
H	3.499931	5.418498	10.22388
C	4.188483	4.891347	12.1244
H	3.319013	5.074687	12.58313
C	4.866138	3.667012	12.76294
H	5.027716	3.836876	13.72476
H	4.275091	2.876862	12.68273
C	6.19822	3.393588	12.05272
H	6.631672	2.58902	12.45866
C	5.931826	3.131816	10.56252
H	5.347601	2.338169	10.46817
H	6.78816	2.93707	10.10546
C	6.192447	5.559403	10.07958
H	5.786533	6.362172	9.666193
H	7.054843	5.383533	9.626344
C	6.44369	5.82395	11.57954
H	7.040792	6.620087	11.67769
C	5.124238	6.098831	12.28197
H	4.699544	6.903322	11.89141
H	5.287472	6.271327	13.24306
C	7.120822	4.604047	12.21546
H	7.988732	4.429725	11.77226
H	7.28989	4.773633	13.17607
C	9.674114	-0.66415	5.671998
C	10.83899	-0.51404	6.307297
H	11.62528	-0.60106	5.781307
C	11.03305	-0.22414	7.75616
H	11.47926	-0.88751	8.269299
C	10.63641	0.891078	8.413564
C	9.901915	1.992338	7.728235
H	10.30728	2.851218	7.705738
C	8.7108	1.847627	7.144286
H	8.31254	2.628526	6.778112
C	7.95486	0.586644	7.014673
H	7.08893	0.558027	7.404357

C	8.381665	-0.51566	6.398299
H	7.81063	-1.27443	6.424481
C	9.569828	-1.03604	4.180727
C	8.921158	0.14006	3.44357
H	8.026663	0.316788	3.829289
H	9.474162	0.95166	3.568431
C	8.780993	-0.1539	1.950331
H	8.355963	0.628421	1.494983
C	10.13269	-0.42356	1.331483
H	10.70661	0.377886	1.423104
H	10.02445	-0.61684	0.366596
C	10.79704	-1.6124	2.021845
H	11.69833	-1.77526	1.620378
C	10.94635	-1.29628	3.531282
H	11.38626	-2.05804	3.985505
H	11.52077	-0.4981	3.645513
C	8.69879	-2.30902	3.972879
H	9.128405	-3.08147	4.418806
H	7.808416	-2.17252	4.383598
C	8.534346	-2.60594	2.471962
H	7.958545	-3.41466	2.351826
C	7.887058	-1.40534	1.768524
H	6.990908	-1.23524	2.153342
H	7.777803	-1.60138	0.804312
C	9.919573	-2.86273	1.851089
H	10.34813	-3.63617	2.296313
H	9.821482	-3.07437	0.888958
C	10.96876	1.126104	9.886415
C	9.657143	1.404308	10.62636
H	9.05145	0.627969	10.52374
H	9.214231	2.192948	10.22388
C	9.902783	1.665797	12.1244
H	9.033313	1.849137	12.58313
C	10.58044	0.441462	12.76294
H	10.74202	0.611326	13.72476
H	9.989391	-0.34869	12.68273
C	11.91252	0.168038	12.05272
H	12.34597	-0.63653	12.45866
C	11.64613	-0.09373	10.56252
H	11.0619	-0.88738	10.46817
H	12.50246	-0.28848	10.10546
C	11.90675	2.333853	10.07958
H	11.50083	3.136622	9.666193
H	12.76914	2.157983	9.626344
C	12.15799	2.5984	11.57954
H	12.75509	3.394537	11.67769
C	10.83854	2.873281	12.28197

H	10.41384	3.677772	11.89141
H	11.00177	3.045777	13.24306
C	12.83512	1.378497	12.21546
H	13.70303	1.204175	11.77226
H	13.00419	1.548083	13.17607
C	3.959814	9.012496	5.671998
C	5.124688	9.162607	6.307297
H	5.91098	9.075595	5.781307
C	5.31875	9.452513	7.75616
H	5.764958	8.78914	8.269299
C	4.922108	10.56773	8.413564
C	4.187615	11.66899	7.728235
H	4.592983	12.52787	7.705738
C	2.9965	11.52428	7.144286
H	2.59824	12.30518	6.778112
C	2.24056	10.26329	7.014673
H	1.37463	10.23468	7.404357
C	2.667365	9.160988	6.398299
H	2.09633	8.402216	6.424481
C	3.855528	8.64061	4.180727
C	3.206858	9.81671	3.44357
H	2.312363	9.993438	3.829289
H	3.759862	10.62831	3.568431
C	3.066693	9.522753	1.950331
H	2.641663	10.30507	1.494983
C	4.418385	9.25309	1.331483
H	4.99231	10.05454	1.423104
H	4.310148	9.059815	0.366596
C	5.082741	8.064249	2.021845
H	5.984031	7.901391	1.620378
C	5.232054	8.380366	3.531282
H	5.671955	7.618607	3.985505
H	5.806469	9.178554	3.645513
C	2.98449	7.367634	3.972879
H	3.414105	6.595185	4.418806
H	2.094116	7.504132	4.383598
C	2.820046	7.070709	2.471962
H	2.244245	6.261992	2.351826
C	2.172758	8.27131	1.768524
H	1.276608	8.441413	2.153342
H	2.063503	8.075274	0.804312
C	4.205273	6.813916	1.851089
H	4.633831	6.040481	2.296313
H	4.107182	6.602282	0.888958
C	5.254459	10.80275	9.886415
C	3.942843	11.08096	10.62636
H	3.33715	10.30462	10.52374

H	3.499931	11.8696	10.22388
C	4.188483	11.34245	12.1244
H	3.319013	11.52579	12.58313
C	4.866138	10.11811	12.76294
H	5.027716	10.28798	13.72476
H	4.275091	9.327962	12.68273
C	6.19822	9.844688	12.05272
H	6.631672	9.04012	12.45866
C	5.931826	9.582916	10.56252
H	5.347601	8.789269	10.46817
H	6.78816	9.38817	10.10546
C	6.192447	12.0105	10.07958
H	5.786533	12.81327	9.666193
H	7.054843	11.83463	9.626344
C	6.44369	12.27505	11.57954
H	7.040792	13.07119	11.67769
C	5.124238	12.54993	12.28197
H	4.699544	13.35442	11.89141
H	5.287472	12.72243	13.24306
C	7.120822	11.05515	12.21546
H	7.988732	10.88083	11.77226
H	7.28989	11.22473	13.17607
C	-1.75449	5.786946	5.671998
C	-0.58961	5.937057	6.307297
H	0.19668	5.850045	5.781307
C	-0.39555	6.226963	7.75616
H	0.050658	5.56359	8.269299
C	-0.79219	7.342178	8.413564
C	-1.52669	8.443438	7.728235
H	-1.12132	9.302318	7.705738
C	-2.7178	8.298727	7.144286
H	-3.11606	9.079626	6.778112
C	-3.47374	7.037744	7.014673
H	-4.33967	7.009127	7.404357
C	-3.04694	5.935438	6.398299
H	-3.61797	5.176666	6.424481
C	-1.85877	5.41506	4.180727
C	-2.50744	6.59116	3.44357
H	-3.40194	6.767888	3.829289
H	-1.95444	7.40276	3.568431
C	-2.64761	6.297203	1.950331
H	-3.07264	7.079521	1.494983
C	-1.29592	6.02754	1.331483
H	-0.72199	6.828986	1.423104
H	-1.40415	5.834265	0.366596
C	-0.63156	4.838699	2.021845
H	0.269731	4.675841	1.620378

C	-0.48225	5.154816	3.531282
H	-0.04235	4.393057	3.985505
H	0.092169	5.953004	3.645513
C	-2.72981	4.142084	3.972879
H	-2.3002	3.369635	4.418806
H	-3.62018	4.278582	4.383598
C	-2.89425	3.845159	2.471962
H	-3.47006	3.036442	2.351826
C	-3.54154	5.04576	1.768524
H	-4.43769	5.215863	2.153342
H	-3.6508	4.849724	0.804312
C	-1.50903	3.588366	1.851089
H	-1.08047	2.814931	2.296313
H	-1.60712	3.376732	0.888958
C	-0.45984	7.577204	9.886415
C	-1.77146	7.855408	10.62636
H	-2.37715	7.079069	10.52374
H	-2.21437	8.644048	10.22388
C	-1.52582	8.116897	12.1244
H	-2.39529	8.300237	12.58313
C	-0.84816	6.892562	12.76294
H	-0.68658	7.062426	13.72476
H	-1.43921	6.102412	12.68273
C	0.48392	6.619138	12.05272
H	0.917372	5.81457	12.45866
C	0.217526	6.357366	10.56252
H	-0.3667	5.563719	10.46817
H	1.07386	6.16262	10.10546
C	0.478147	8.784953	10.07958
H	0.072233	9.587722	9.666193
H	1.340543	8.609083	9.626344
C	0.72939	9.0495	11.57954
H	1.326492	9.845637	11.67769
C	-0.59006	9.324381	12.28197
H	-1.01476	10.12887	11.89141
H	-0.42683	9.496877	13.24306
C	1.406522	7.829597	12.21546
H	2.274432	7.655275	11.77226
H	1.57559	7.999183	13.17607
C	-1.75449	-0.66415	5.671998
C	-0.58961	-0.51404	6.307297
H	0.19668	-0.60106	5.781307
C	-0.39555	-0.22414	7.75616
H	0.050658	-0.88751	8.269299
C	-0.79219	0.891078	8.413564
C	-1.52669	1.992338	7.728235
H	-1.12132	2.851218	7.705738

C	-2.7178	1.847627	7.144286
H	-3.11606	2.628526	6.778112
C	-3.47374	0.586644	7.014673
H	-4.33967	0.558027	7.404357
C	-3.04694	-0.51566	6.398299
H	-3.61797	-1.27443	6.424481
C	-1.85877	-1.03604	4.180727
C	-2.50744	0.14006	3.44357
H	-3.40194	0.316788	3.829289
H	-1.95444	0.95166	3.568431
C	-2.64761	-0.1539	1.950331
H	-3.07264	0.628421	1.494983
C	-1.29592	-0.42356	1.331483
H	-0.72199	0.377886	1.423104
H	-1.40415	-0.61684	0.366596
C	-0.63156	-1.6124	2.021845
H	0.269731	-1.77526	1.620378
C	-0.48225	-1.29628	3.531282
H	-0.04235	-2.05804	3.985505
H	0.092169	-0.4981	3.645513
C	-2.72981	-2.30902	3.972879
H	-2.3002	-3.08147	4.418806
H	-3.62018	-2.17252	4.383598
C	-2.89425	-2.60594	2.471962
H	-3.47006	-3.41466	2.351826
C	-3.54154	-1.40534	1.768524
H	-4.43769	-1.23524	2.153342
H	-3.6508	-1.60138	0.804312
C	-1.50903	-2.86273	1.851089
H	-1.08047	-3.63617	2.296313
H	-1.60712	-3.07437	0.888958
C	-0.45984	1.126104	9.886415
C	-1.77146	1.404308	10.62636
H	-2.37715	0.627969	10.52374
H	-2.21437	2.192948	10.22388
C	-1.52582	1.665797	12.1244
H	-2.39529	1.849137	12.58313
C	-0.84816	0.441462	12.76294
H	-0.68658	0.611326	13.72476
H	-1.43921	-0.34869	12.68273
C	0.48392	0.168038	12.05272
H	0.917372	-0.63653	12.45866
C	0.217526	-0.09373	10.56252
H	-0.3667	-0.88738	10.46817
H	1.07386	-0.28848	10.10546
C	0.478147	2.333853	10.07958
H	0.072233	3.136622	9.666193

H	1.340543	2.157983	9.626344
C	0.72939	2.5984	11.57954
H	1.326492	3.394537	11.67769
C	-0.59006	2.873281	12.28197
H	-1.01476	3.677772	11.89141
H	-0.42683	3.045777	13.24306
C	1.406522	1.378497	12.21546
H	2.274432	1.204175	11.77226
H	1.57559	1.548083	13.17607
C	9.674114	5.786946	5.671998
C	10.83899	5.937057	6.307297
H	11.62528	5.850045	5.781307
C	11.03305	6.226963	7.75616
H	11.47926	5.56359	8.269299
C	10.63641	7.342178	8.413564
C	9.901915	8.443438	7.728235
H	10.30728	9.302318	7.705738
C	8.7108	8.298727	7.144286
H	8.31254	9.079626	6.778112
C	7.95486	7.037744	7.014673
H	7.08893	7.009127	7.404357
C	8.381665	5.935438	6.398299
H	7.81063	5.176666	6.424481
C	9.569828	5.41506	4.180727
C	8.921158	6.59116	3.44357
H	8.026663	6.767888	3.829289
H	9.474162	7.40276	3.568431
C	8.780993	6.297203	1.950331
H	8.355963	7.079521	1.494983
C	10.13269	6.02754	1.331483
H	10.70661	6.828986	1.423104
H	10.02445	5.834265	0.366596
C	10.79704	4.838699	2.021845
H	11.69833	4.675841	1.620378
C	10.94635	5.154816	3.531282
H	11.38626	4.393057	3.985505
H	11.52077	5.953004	3.645513
C	8.69879	4.142084	3.972879
H	9.128405	3.369635	4.418806
H	7.808416	4.278582	4.383598
C	8.534346	3.845159	2.471962
H	7.958545	3.036442	2.351826
C	7.887058	5.04576	1.768524
H	6.990908	5.215863	2.153342
H	7.777803	4.849724	0.804312
C	9.919573	3.588366	1.851089
H	10.34813	2.814931	2.296313

H	9.821482	3.376732	0.888958
C	10.96876	7.577204	9.886415
C	9.657143	7.855408	10.62636
H	9.05145	7.079069	10.52374
H	9.214231	8.644048	10.22388
C	9.902783	8.116897	12.1244
H	9.033313	8.300237	12.58313
C	10.58044	6.892562	12.76294
H	10.74202	7.062426	13.72476
H	9.989391	6.102412	12.68273
C	11.91252	6.619138	12.05272
H	12.34597	5.81457	12.45866
C	11.64613	6.357366	10.56252
H	11.0619	5.563719	10.46817
H	12.50246	6.16262	10.10546
C	11.90675	8.784953	10.07958
H	11.50083	9.587722	9.666193
H	12.76914	8.609083	9.626344
C	12.15799	9.0495	11.57954
H	12.75509	9.845637	11.67769
C	10.83854	9.324381	12.28197
H	10.41384	10.12887	11.89141
H	11.00177	9.496877	13.24306
C	12.83512	7.829597	12.21546
H	13.70303	7.655275	11.77226
H	13.00419	7.999183	13.17607
C	3.959814	-3.8897	5.671998
C	5.124688	-3.73959	6.307297
H	5.91098	-3.82661	5.781307
C	5.31875	-3.44969	7.75616
H	5.764958	-4.11306	8.269299
C	4.922108	-2.33447	8.413564
C	4.187615	-1.23321	7.728235
H	4.592983	-0.37433	7.705738
C	2.9965	-1.37792	7.144286
H	2.59824	-0.59702	6.778112
C	2.24056	-2.63891	7.014673
H	1.37463	-2.66752	7.404357
C	2.667365	-3.74121	6.398299
H	2.09633	-4.49998	6.424481
C	3.855528	-4.26159	4.180727
C	3.206858	-3.08549	3.44357
H	2.312363	-2.90876	3.829289
H	3.759862	-2.27389	3.568431
C	3.066693	-3.37945	1.950331
H	2.641663	-2.59713	1.494983
C	4.418385	-3.64911	1.331483

H	4.99231	-2.84766	1.423104
H	4.310148	-3.84239	0.366596
C	5.082741	-4.83795	2.021845
H	5.984031	-5.00081	1.620378
C	5.232054	-4.52183	3.531282
H	5.671955	-5.28359	3.985505
H	5.806469	-3.72365	3.645513
C	2.98449	-5.53457	3.972879
H	3.414105	-6.30702	4.418806
H	2.094116	-5.39807	4.383598
C	2.820046	-5.83149	2.471962
H	2.244245	-6.64021	2.351826
C	2.172758	-4.63089	1.768524
H	1.276608	-4.46079	2.153342
H	2.063503	-4.82693	0.804312
C	4.205273	-6.08828	1.851089
H	4.633831	-6.86172	2.296313
H	4.107182	-6.29992	0.888958
C	5.254459	-2.09945	9.886415
C	3.942843	-1.82124	10.62636
H	3.33715	-2.59758	10.52374
H	3.499931	-1.0326	10.22388
C	4.188483	-1.55975	12.1244
H	3.319013	-1.37641	12.58313
C	4.866138	-2.78409	12.76294
H	5.027716	-2.61422	13.72476
H	4.275091	-3.57424	12.68273
C	6.19822	-3.05751	12.05272
H	6.631672	-3.86208	12.45866
C	5.931826	-3.31928	10.56252
H	5.347601	-4.11293	10.46817
H	6.78816	-3.51403	10.10546
C	6.192447	-0.8917	10.07958
H	5.786533	-0.08893	9.666193
H	7.054843	-1.06757	9.626344
C	6.44369	-0.62715	11.57954
H	7.040792	0.168987	11.67769
C	5.124238	-0.35227	12.28197
H	4.699544	0.452222	11.89141
H	5.287472	-0.17977	13.24306
C	7.120822	-1.84705	12.21546
H	7.988732	-2.02138	11.77226
H	7.28989	-1.67747	13.17607
C	1.754486	5.786946	-5.672
C	0.589612	5.937057	-6.3073
H	-0.19668	5.850045	-5.78131
C	0.39555	6.226963	-7.75616

H	-0.05066	5.56359	-8.2693
C	0.792192	7.342178	-8.41356
C	1.526685	8.443438	-7.72824
H	1.121317	9.302318	-7.70574
C	2.7178	8.298727	-7.14429
H	3.11606	9.079626	-6.77811
C	3.47374	7.037744	-7.01467
H	4.33967	7.009127	-7.40436
C	3.046935	5.935438	-6.3983
H	3.61797	5.176666	-6.42448
C	1.858772	5.41506	-4.18073
C	2.507442	6.59116	-3.44357
H	3.401937	6.767888	-3.82929
H	1.954438	7.40276	-3.56843
C	2.647607	6.297203	-1.95033
H	3.072637	7.079521	-1.49498
C	1.295915	6.02754	-1.33148
H	0.72199	6.828986	-1.4231
H	1.404152	5.834265	-0.3666
C	0.631559	4.838699	-2.02185
H	-0.26973	4.675841	-1.62038
C	0.482246	5.154816	-3.53128
H	0.042345	4.393057	-3.98551
H	-0.09217	5.953004	-3.64551
C	2.72981	4.142084	-3.97288
H	2.300195	3.369635	-4.41881
H	3.620184	4.278582	-4.3836
C	2.894254	3.845159	-2.47196
H	3.470055	3.036442	-2.35183
C	3.541542	5.04576	-1.76852
H	4.437692	5.215863	-2.15334
H	3.650797	4.849724	-0.80431
C	1.509027	3.588366	-1.85109
H	1.080469	2.814931	-2.29631
H	1.607118	3.376732	-0.88896
C	0.459841	7.577204	-9.88642
C	1.771457	7.855408	-10.6264
H	2.37715	7.079069	-10.5237
H	2.214369	8.644048	-10.2239
C	1.525817	8.116897	-12.1244
H	2.395287	8.300237	-12.5831
C	0.848162	6.892562	-12.7629
H	0.686584	7.062426	-13.7248
H	1.439209	6.102412	-12.6827
C	-0.48392	6.619138	-12.0527
H	-0.91737	5.81457	-12.4587
C	-0.21753	6.357366	-10.5625

H	0.366699	5.563719	-10.4682
H	-1.07386	6.16262	-10.1055
C	-0.47815	8.784953	-10.0796
H	-0.07223	9.587722	-9.66619
H	-1.34054	8.609083	-9.62634
C	-0.72939	9.0495	-11.5795
H	-1.32649	9.845637	-11.6777
C	0.590062	9.324381	-12.282
H	1.014756	10.12887	-11.8914
H	0.426828	9.496877	-13.2431
C	-1.40652	7.829597	-12.2155
H	-2.27443	7.655275	-11.7723
H	-1.57559	7.999183	-13.1761
C	1.754486	-0.66415	-5.672
C	0.589612	-0.51404	-6.3073
H	-0.19668	-0.60106	-5.78131
C	0.39555	-0.22414	-7.75616
H	-0.05066	-0.88751	-8.2693
C	0.792192	0.891078	-8.41356
C	1.526685	1.992338	-7.72824
H	1.121317	2.851218	-7.70574
C	2.7178	1.847627	-7.14429
H	3.11606	2.628526	-6.77811
C	3.47374	0.586644	-7.01467
H	4.33967	0.558027	-7.40436
C	3.046935	-0.51566	-6.3983
H	3.61797	-1.27443	-6.42448
C	1.858772	-1.03604	-4.18073
C	2.507442	0.14006	-3.44357
H	3.401937	0.316788	-3.82929
H	1.954438	0.95166	-3.56843
C	2.647607	-0.1539	-1.95033
H	3.072637	0.628421	-1.49498
C	1.295915	-0.42356	-1.33148
H	0.72199	0.377886	-1.4231
H	1.404152	-0.61684	-0.3666
C	0.631559	-1.6124	-2.02185
H	-0.26973	-1.77526	-1.62038
C	0.482246	-1.29628	-3.53128
H	0.042345	-2.05804	-3.98551
H	-0.09217	-0.4981	-3.64551
C	2.72981	-2.30902	-3.97288
H	2.300195	-3.08147	-4.41881
H	3.620184	-2.17252	-4.3836
C	2.894254	-2.60594	-2.47196
H	3.470055	-3.41466	-2.35183
C	3.541542	-1.40534	-1.76852

H	4.437692	-1.23524	-2.15334
H	3.650797	-1.60138	-0.80431
C	1.509027	-2.86273	-1.85109
H	1.080469	-3.63617	-2.29631
H	1.607118	-3.07437	-0.88896
C	0.459841	1.126104	-9.88642
C	1.771457	1.404308	-10.6264
H	2.37715	0.627969	-10.5237
H	2.214369	2.192948	-10.2239
C	1.525817	1.665797	-12.1244
H	2.395287	1.849137	-12.5831
C	0.848162	0.441462	-12.7629
H	0.686584	0.611326	-13.7248
H	1.439209	-0.34869	-12.6827
C	-0.48392	0.168038	-12.0527
H	-0.91737	-0.63653	-12.4587
C	-0.21753	-0.09373	-10.5625
H	0.366699	-0.88738	-10.4682
H	-1.07386	-0.28848	-10.1055
C	-0.47815	2.333853	-10.0796
H	-0.07223	3.136622	-9.66619
H	-1.34054	2.157983	-9.62634
C	-0.72939	2.5984	-11.5795
H	-1.32649	3.394537	-11.6777
C	0.590062	2.873281	-12.282
H	1.014756	3.677772	-11.8914
H	0.426828	3.045777	-13.2431
C	-1.40652	1.378497	-12.2155
H	-2.27443	1.204175	-11.7723
H	-1.57559	1.548083	-13.1761
C	8.845181	5.786946	22.4498
C	7.680306	5.937057	21.8145
H	6.894014	5.850045	22.34049
C	7.486244	6.226963	20.36564
H	7.040037	5.56359	19.8525
C	7.882887	7.342178	19.70823
C	8.61738	8.443438	20.39356
H	8.212011	9.302318	20.41606
C	9.808495	8.298727	20.97751
H	10.20676	9.079626	21.34368
C	10.56444	7.037744	21.10712
H	11.43036	7.009127	20.71744
C	10.13763	5.935438	21.7235
H	10.70866	5.176666	21.69732
C	8.949467	5.41506	23.94107
C	9.598136	6.59116	24.67823
H	10.49263	6.767888	24.29251

H	9.045132	7.40276	24.55337
C	9.738301	6.297203	26.17147
H	10.16333	7.079521	26.62681
C	8.38661	6.02754	26.79031
H	7.812684	6.828986	26.69869
H	8.494847	5.834265	27.7552
C	7.722253	4.838699	26.09995
H	6.820963	4.675841	26.50142
C	7.572941	5.154816	24.59052
H	7.13304	4.393057	24.13629
H	6.998526	5.953004	24.47628
C	9.820505	4.142084	24.14892
H	9.390889	3.369635	23.70299
H	10.71088	4.278582	23.7382
C	9.984949	3.845159	25.64984
H	10.56075	3.036442	25.76997
C	10.63224	5.04576	26.35327
H	11.52839	5.215863	25.96846
H	10.74149	4.849724	27.31749
C	8.599722	3.588366	26.27071
H	8.171164	2.814931	25.82548
H	8.697812	3.376732	27.23284
C	7.550536	7.577204	18.23538
C	8.862152	7.855408	17.49544
H	9.467845	7.079069	17.59806
H	9.305063	8.644048	17.89792
C	8.616511	8.116897	15.99739
H	9.485982	8.300237	15.53867
C	7.938857	6.892562	15.35886
H	7.777278	7.062426	14.39704
H	8.529904	6.102412	15.43906
C	6.606774	6.619138	16.06908
H	6.173322	5.81457	15.66314
C	6.873169	6.357366	17.55928
H	7.457394	5.563719	17.65363
H	6.016834	6.16262	18.01634
C	6.612548	8.784953	18.04221
H	7.018462	9.587722	18.4556
H	5.750152	8.609083	18.49545
C	6.361304	9.0495	16.54225
H	5.764202	9.845637	16.44411
C	7.680757	9.324381	15.83983
H	8.10545	10.12887	16.23038
H	7.517523	9.496877	14.87874
C	5.684173	7.829597	15.90634
H	4.816262	7.655275	16.34954
H	5.515105	7.999183	14.94572

C	8.845181	-0.66415	22.4498
C	7.680306	-0.51404	21.8145
H	6.894014	-0.60106	22.34049
C	7.486244	-0.22414	20.36564
H	7.040037	-0.88751	19.8525
C	7.882887	0.891078	19.70823
C	8.61738	1.992338	20.39356
H	8.212011	2.851218	20.41606
C	9.808495	1.847627	20.97751
H	10.20676	2.628526	21.34368
C	10.56444	0.586644	21.10712
H	11.43036	0.558027	20.71744
C	10.13763	-0.51566	21.7235
H	10.70866	-1.27443	21.69732
C	8.949467	-1.03604	23.94107
C	9.598136	0.14006	24.67823
H	10.49263	0.316788	24.29251
H	9.045132	0.95166	24.55337
C	9.738301	-0.1539	26.17147
H	10.16333	0.628421	26.62681
C	8.38661	-0.42356	26.79031
H	7.812684	0.377886	26.69869
H	8.494847	-0.61684	27.7552
C	7.722253	-1.6124	26.09995
H	6.820963	-1.77526	26.50142
C	7.572941	-1.29628	24.59052
H	7.13304	-2.05804	24.13629
H	6.998526	-0.4981	24.47628
C	9.820505	-2.30902	24.14892
H	9.390889	-3.08147	23.70299
H	10.71088	-2.17252	23.7382
C	9.984949	-2.60594	25.64984
H	10.56075	-3.41466	25.76997
C	10.63224	-1.40534	26.35327
H	11.52839	-1.23524	25.96846
H	10.74149	-1.60138	27.31749
C	8.599722	-2.86273	26.27071
H	8.171164	-3.63617	25.82548
H	8.697812	-3.07437	27.23284
C	7.550536	1.126104	18.23538
C	8.862152	1.404308	17.49544
H	9.467845	0.627969	17.59806
H	9.305063	2.192948	17.89792
C	8.616511	1.665797	15.99739
H	9.485982	1.849137	15.53867
C	7.938857	0.441462	15.35886
H	7.777278	0.611326	14.39704

H	8.529904	-0.34869	15.43906
C	6.606774	0.168038	16.06908
H	6.173322	-0.63653	15.66314
C	6.873169	-0.09373	17.55928
H	7.457394	-0.88738	17.65363
H	6.016834	-0.28848	18.01634
C	6.612548	2.333853	18.04221
H	7.018462	3.136622	18.4556
H	5.750152	2.157983	18.49545
C	6.361304	2.5984	16.54225
H	5.764202	3.394537	16.44411
C	7.680757	2.873281	15.83983
H	8.10545	3.677772	16.23038
H	7.517523	3.045777	14.87874
C	5.684173	1.378497	15.90634
H	4.816262	1.204175	16.34954
H	5.515105	1.548083	14.94572

Table S16. Geometric coordinates of **$C_{20}H_{20}$-CH_4** optimized at PBE0-D4/cc-pVTZ level.

Atom	X	Y	Z
C	0.3326781	2.9925746	1.8755676
C	-0.337823	1.8453651	1.0952502
H	0.5788262	2.6807331	2.8914183
H	-0.430038	0.9535893	1.7166226
C	0.3265901	3.0299566	-2.158723
C	1.5965917	3.4371406	-1.386578
H	0.5697742	2.7370818	-3.180911
H	2.4815343	3.349425	-2.018107
C	-0.642761	4.2278474	-2.146016
C	-1.910339	3.8062377	-1.377938
H	-0.889034	4.5398175	-3.161789
H	-2.797058	3.9058526	-2.00519
C	-0.63664	4.1904972	1.8880552
C	0.0312989	5.3522344	1.127156
H	-0.879857	4.4835561	2.9101718
H	0.1260139	6.2319915	1.7649722
C	-1.721931	2.3337411	0.6259147
H	-2.513346	1.6892097	1.0105172
C	1.4139266	4.8723168	0.644549
H	2.206483	5.509687	1.0386592
C	1.4116496	4.886579	-0.896377
H	2.2030462	5.5311803	-1.280924
C	-1.724189	2.3479432	-0.915169
H	-2.516784	1.7106692	-1.309334
C	0.5153709	1.5582633	-0.155311
H	0.8530932	0.5223509	-0.164404
C	-2.023562	4.6930014	-0.122478
H	-2.967208	5.2399051	-0.115965
C	-0.826203	5.6629383	-0.115282
H	-1.164582	6.6998223	-0.105164
C	1.7130478	2.527118	-0.148078
H	2.6557777	1.9781401	-0.155567

C	0.0275488	5.3753162	-1.365852
H	0.1205214	6.2666228	-1.987695
C	-1.906611	3.7831782	1.116023
H	-2.791399	3.8711064	1.7477406
C	1.6003829	3.4139611	1.1074325
H	2.4872367	3.3146617	1.7345889
C	-0.341492	1.8683515	-1.397737
H	-0.435803	0.9882752	-2.035345
C	2.5410202	-1.887385	-0.07748
H	3.397961	-2.533755	-0.261897
H	2.1071306	-1.580241	-1.027909
H	2.8631911	-1.006725	0.4763045
H	1.7961658	-2.429462	0.5033961

Table S17. Geometric coordinates of $\mathbf{C_{20}H_{20}}$-$\mathbf{C_4H_{10}}$ optimized at PBE0-D4/cc-pVTZ level.

Atom	X	Y	Z
C	0.261674	3.370533	2.142557
C	-0.489	2.116855	1.653087
H	0.485536	3.299674	3.207608
H	-0.64385	1.412331	2.471114
C	0.258514	2.423318	-1.77905
C	1.554018	2.917698	-1.10734
H	0.479446	1.874437	-2.69557
H	2.429859	2.616768	-1.68324
C	-0.62396	3.651717	-2.07509
C	-1.91803	3.518359	-1.24903
H	-0.84766	3.722358	-3.14014
H	-2.79494	3.521797	-1.89754
C	-0.62076	4.599086	1.846484
C	0.128096	5.49086	0.83747
H	-0.84215	5.148659	2.762186
H	0.284923	6.490961	1.243369
C	-1.83471	2.56991	1.055219
H	-2.66975	2.094548	1.571236
C	1.473349	4.813957	0.509972
H	2.309095	5.472104	0.750656
C	1.472188	4.45203	-0.98784
H	2.307285	4.927208	-1.50395
C	-1.83574	2.20785	-0.44267
H	-2.67118	1.54944	-0.68352
C	0.341066	1.47569	0.525276
H	0.6039	0.447775	0.775283
C	-1.96783	4.69027	-0.24975
H	-2.87017	5.285827	-0.39342
C	-0.70405	5.547552	-0.45786
H	-0.96804	6.576254	-0.70613
C	1.605134	2.331424	0.316708
H	2.506891	1.734062	0.458734

C	0.12648	4.905751	-1.58579
H	0.281814	5.609577	-2.40434
C	-1.91627	4.104213	1.174599
H	-2.79247	4.403974	1.750705
C	1.555869	3.503285	1.316235
H	2.432974	3.499648	1.964494
C	-0.49027	1.53165	-0.76984
H	-0.6473	0.531235	-1.17623
C	2.513299	-1.58987	-0.41187
C	3.693753	-2.52208	-0.18106
C	2.681726	-0.805	-1.70438
H	2.484029	-0.87216	0.416666
C	1.202111	-2.36221	-0.41203
H	4.636666	-1.9714	-0.14976
H	3.765631	-3.25862	-0.98703
H	3.59198	-3.06873	0.759296
H	1.188155	-3.09473	-1.22476
H	0.34673	-1.69695	-0.5512
H	1.057524	-2.9041	0.525483
H	3.598387	-0.21071	-1.69409
H	1.843059	-0.12622	-1.87092
H	2.734729	-1.4834	-2.56143

Table S18. Geometric coordinates of **$C_{20}H_{20}$-C_5H_{12}** optimized at PBE0-D4/cc-pVTZ level.

Atom	X	Y	Z
C	0.116015	3.557689	2.155485
C	-0.5931	2.272141	1.687103
H	0.295191	3.533585	3.231029
H	-0.77086	1.598386	2.526087
C	0.295389	2.451851	-1.72107
C	1.553008	2.996448	-1.01591
H	0.565744	1.86934	-2.60267
H	2.457406	2.686845	-1.54101
C	-0.59244	3.651624	-2.10374
C	-1.91846	3.529682	-1.32816
H	-0.77167	3.67551	-3.17928
H	-2.76685	3.492068	-2.01255
C	-0.77175	4.757291	1.772372
C	0.005333	5.620633	0.759845
H	-1.04145	5.339451	2.654294
H	0.128451	6.638975	1.130435
C	-1.91859	2.676869	1.014657
H	-2.76737	2.20825	1.514166
C	1.373603	4.954743	0.517238
H	2.187943	5.636464	0.76549
C	1.442061	4.532277	-0.96322

H	2.29093	5.000686	-1.46277
C	-1.84991	2.254534	-0.46571
H	-2.66403	1.57263	-0.71408
C	0.294724	1.600641	0.622273
H	0.563726	0.587376	0.924271
C	-2.02939	4.740012	-0.38092
H	-2.93392	5.313904	-0.5861
C	-0.77153	5.610238	-0.57087
H	-1.04083	6.623194	-0.87235
C	1.552476	2.470068	0.432498
H	2.457572	1.896365	0.635814
C	0.116548	4.937432	-1.63554
H	0.294967	5.610337	-2.4751
C	-2.02949	4.212939	1.067344
H	-2.93428	4.52052	1.592872
C	1.442	3.679717	1.379779
H	2.290587	3.716526	2.063927
C	-0.48171	1.588626	-0.70848
H	-0.60465	0.569741	-1.07868
C	2.799982	-1.88375	-0.41181
C	3.741307	-2.98936	-0.88045
C	2.72216	-0.79472	-1.47836
C	1.407713	-2.46483	-0.18001
H	4.747081	-2.59962	-1.05757
H	3.386933	-3.43685	-1.81259
H	3.817622	-3.78524	-0.13496
H	1.012745	-2.91313	-1.09535
H	0.706943	-1.68976	0.140907
H	1.427892	-3.23917	0.59114
H	3.703956	-0.34836	-1.65677
H	2.042566	0.003165	-1.17399
H	2.360686	-1.19985	-2.42701
C	3.32684	-1.28675	0.890313
H	2.674532	-0.48769	1.251487
H	4.32574	-0.86494	0.751895
H	3.389699	-2.04668	1.673405

Table S19. Geometric coordinates of $\mathbf{C_{20}H_{20}}$**-**$\mathbf{C_8H_{10}}$ optimized at PBE0-D4/cc-pVTZ level.

Atom	X	Y	Z
C	0.477016	2.862949	1.960012
C	-0.01103	1.582489	1.256274
H	0.714906	2.666443	3.006308
H	-0.0208	0.735458	1.941637
C	0.645431	2.578169	-2.06067
C	1.819832	3.203209	-1.2829
H	0.968241	2.237842	-3.04552

H	2.735885	3.178812	-1.87449
C	-0.46443	3.640224	-2.18284
C	-1.70288	3.123032	-1.42475
H	-0.70246	3.837263	-3.22894
H	-2.56634	3.058691	-2.08822
C	-0.6329	3.925189	1.838367
C	-0.08032	5.097864	1.005227
H	-0.95599	4.266076	2.822858
H	-0.1246	6.030882	1.568396
C	-1.42135	1.851745	0.700984
H	-2.13954	1.142243	1.111772
C	1.371141	4.760587	0.612009
H	2.060214	5.523188	0.977264
C	1.435539	4.651801	-0.92386
H	2.157087	5.359279	-1.3345
C	-1.35784	1.741917	-0.8341
H	-2.04518	0.97661	-1.19684
C	0.924327	1.306875	0.064945
H	1.377716	0.320552	0.156511
C	-1.98007	4.086168	-0.25406
H	-2.98334	4.508156	-0.32611
C	-0.91283	5.19764	-0.28785
H	-1.37712	6.180587	-0.37718
C	1.9925	2.416389	0.031226
H	2.99606	1.99467	0.103086
C	0.023933	4.921779	-1.4797
H	0.032813	5.765162	-2.17141
C	-1.80674	3.29978	1.060001
H	-2.72309	3.324965	1.651257
C	1.715729	3.379513	1.202254
H	2.579261	3.444776	1.865506
C	0.09296	1.406072	-1.2272
H	0.136149	0.470997	-1.78544
H	0.868793	-2.35687	2.446482
C	0.231602	-2.27103	1.572474
C	-1.11736	-2.01767	1.737116
H	-1.52126	-1.90951	2.738365
C	-1.96767	-1.89398	0.639733
C	-3.42932	-1.62339	0.826218
C	-1.41227	-2.03485	-0.62397
H	-2.04779	-1.94164	-1.49803
C	-0.05808	-2.29049	-0.78954
H	0.34673	-2.39293	-1.79064
C	0.789167	-2.41156	0.302704
C	2.254003	-2.67609	0.130841
H	2.524569	-2.72884	-0.924
H	2.544496	-3.61921	0.600387

H	2.855159	-1.8881	0.591869
H	-3.93338	-1.49522	-0.13195
H	-3.59303	-0.71799	1.415722
H	-3.91873	-2.44479	1.355869

Table S20. Geometric coordinates of **Buckminsterfullerene and CH_4** optimized at PBE0-D4/cc-pVTZ level.

Atom	X	Y	Z
C	-5.8076	2.756689	3.305662
C	-6.89522	1.810497	3.303596
C	-6.3321	0.484895	3.369418
C	-4.89637	0.612163	3.411679
C	-4.57234	2.016274	3.372245
C	-3.46527	2.451315	2.665054
C	-2.63648	1.500211	1.967012
C	-2.94794	0.152594	2.00471
C	-4.10073	-0.30056	2.742053
C	-4.70741	-1.37889	2.0017
C	-6.08509	-1.50072	1.961068
C	-6.91434	-0.54988	2.659043
C	-8.08424	-0.30249	1.853347
C	-8.62468	0.969939	1.790389
C	-8.01778	2.048277	2.53043
C	-8.09954	3.242307	1.726333
C	-8.75707	2.90172	0.489193
C	-9.0815	1.497307	0.528673
C	-8.97944	0.73109	-0.61917
C	-8.41645	-0.59433	-0.55338
C	-7.97784	-1.10054	0.65743
C	-6.74234	-1.84115	0.724154
C	-5.99573	-2.04496	-0.42264
C	-4.56049	-1.91809	-0.38037
C	-3.92939	-1.59161	0.806823
C	-2.84139	-0.64577	0.808854
C	-2.42872	-0.06426	-0.37678
C	-2.10411	1.340065	-0.4163
C	-2.20618	2.106387	0.731537
C	-2.76918	3.431819	0.665753
C	-3.20785	3.938019	-0.54502
C	-3.10142	3.139958	-1.7409
C	-2.56088	1.867588	-1.67804
C	-3.16781	0.78923	-2.41807
C	-3.08604	-0.40483	-1.61403
C	-4.13034	-1.31263	-1.61577
C	-5.29971	-1.06507	-2.42172
C	-6.4529	-1.51787	-1.68425
C	-7.63855	-0.80755	-1.74857

C	-7.72031	0.386249	-2.55271
C	-8.54909	1.337305	-1.85465
C	-8.23759	2.684849	-1.89233
C	-8.34401	3.483245	-0.69648
C	-7.25658	4.429868	-0.6945
C	-6.47823	4.216633	-1.88947
C	-7.08477	3.138108	-2.6296
C	-6.28914	2.225411	-3.29937
C	-6.61323	0.821305	-3.25988
C	-5.37795	0.080923	-3.19333
C	-4.29032	1.027048	-3.1913
C	-4.85347	2.352656	-3.2571
C	-4.27131	3.387399	-2.54665
C	-5.10057	4.338267	-1.84872
C	-4.44335	4.678669	-0.61165
C	-5.18988	4.882899	0.535113
C	-6.6254	4.756346	0.492869
C	-7.05555	4.150523	1.728362
C	-5.88592	3.902756	2.53418
C	-4.73279	4.355437	1.796574
C	-3.54706	3.645121	1.860951
C	-1.37433	-3.91598	-0.6742
H	-0.71867	-4.72406	-0.995
H	-1.27245	-3.07415	-1.35763
H	-1.10015	-3.6024	0.331963
H	-2.40693	-4.26123	-0.67661

References

1. Neese, F. Software update: The ORCA program system-Version 5.0. *WIREs Comput. Mol. Sci.* **12**, e1606 (2022).
2. Neese, F. The SHARK integral generation and digestion system. *J. Comput. Chem.* **44**, 381–396 (2023).
3. Becke, A. D. Density-functional thermochemistry. III. The role of exact exchange. *J. Chem. Phys.* **98**, 5648–5652 (1993).
4. Lee, C., Yang, W. & Parr, R. G. Development of the Colle-Salvetti correlation-energy formula into a functional of the electron density. *Phys. Rev. B* **37**, 785–789 (1988).
5. Vosko, S. H., Wilk, L. & Nusair, M. Accurate spin-dependent electron liquid correlation energies for local spin density calculations: a critical analysis. *Can. J. Phys.* **58**, 1200–1211 (1980).

6. Stephens, P. J., Devlin, F. J., Chabalowski, C. F. & Frisch, M. J. Ab Initio Calculation of Vibrational Absorption and Circular Dichroism Spectra Using Density Functional Force Fields. *J. Phys. Chem.* **98**, 11623–11627 (1994).

7. Weigend, F. & Ahlrichs, R. Balanced basis sets of split valence, triple zeta valence and quadruple zeta valence quality for H to Rn: Design and assessment of accuracy. *Phys. Chem. Chem. Phys.* **7**, 3297 (2005).

8. Grimme, S., Antony, J., Ehrlich, S. & Krieg, H. A consistent and accurate *ab initio* parametrization of density functional dispersion correction (DFT-D) for the 94 elements H-Pu. *J. Chem. Phys.* **132**, 154104 (2010).

9. Caldeweyher, E., Bannwarth, C. & Grimme, S. Extension of the D3 dispersion coefficient model. *J. Chem. Phys.* **147**, 034112 (2017).

10. Caldeweyher, E. *et al.* A generally applicable atomic-charge dependent London dispersion correction. *J. Chem. Phys.* **150**, 154122 (2019).

11. Grimme, S. Density functional theory with London dispersion corrections. *WIREs Comput. Mol. Sci.* **1**, 211–228 (2011).

12. Grimme, S., Ehrlich, S. & Goerigk, L. Effect of the damping function in dispersion corrected density functional theory. *J. Comput. Chem.* **32**, 1456–1465 (2011).

13. Smith, D. G. A., Burns, L. A., Patkowski, K. & Sherrill, C. D. Revised Damping Parameters for the D3 Dispersion Correction to Density Functional Theory. *J. Phys. Chem. Lett.* **7**, 2197–2203 (2016).

14. Bartlett, R. J. & Musiał, M. Coupled-cluster theory in quantum chemistry. *Rev. Mod. Phys.* **79**, 291–352 (2007).

15. Riplinger, C., Pinski, P., Becker, U., Valeev, E. F. & Neese, F. Sparse maps—A systematic infrastructure for reduced-scaling electronic structure methods. II. Linear scaling domain based pair natural orbital coupled cluster theory. *J. Chem. Phys.* **144**, 024109 (2016).

16. Riplinger, C., Sandhoefer, B., Hansen, A. & Neese, F. Natural triple excitations in local coupled cluster calculations with pair natural orbitals. *J. Chem. Phys.* **139**, 134101 (2013).

17. Saitow, M., Becker, U., Riplinger, C., Valeev, E. F. & Neese, F. A new near-linear scaling, efficient and accurate, open-shell domain-based local pair natural orbital coupled cluster singles and doubles theory. *J. Chem. Phys.* **146**, 164105 (2017).
18. Schneider, W. B. *et al.* Decomposition of Intermolecular Interaction Energies within the Local Pair Natural Orbital Coupled Cluster Framework. *J. Chem. Theory Comput.* **12**, 4778–4792 (2016).
19. Altun, A., Izsák, R. & Bistoni, G. Local energy decomposition of coupled-cluster interaction energies: Interpretation, benchmarks, and comparison with symmetry-adapted perturbation theory. *Int. J. Quantum Chem.* **121**, e26339 (2021).
20. Bistoni, G., Altun, A., Wang, Z. & Neese, F. Local Energy Decomposition Analysis of London Dispersion Effects: From Simple Model Dimers to Complex Biomolecular Assemblies. *Acc. Chem. Res.* **57**, 1411–1420 (2024).
21. Dunning, T. H. Gaussian basis sets for use in correlated molecular calculations. I. The atoms boron through neon and hydrogen. *J. Chem. Phys.* **90**, 1007–1023 (1989).
22. Stoychev, G. L., Auer, A. A. & Neese, F. Automatic Generation of Auxiliary Basis Sets. *J. Chem. Theory Comput.* **13**, 554–562 (2017).
23. Adamo, C., Cossi, M. & Barone, V. An accurate density functional method for the study of magnetic properties: the PBE0 model. *J. Mol. Struct. THEOCHEM* **493**, 145–157 (1999).
24. Regni, G. & Bistoni, G. bistonigroup/LDDSuite: codes for London dispersion density (difference) function. https://github.com/bistonigroup/LDDsuite.
25. Boys, S. F. Construction of Some Molecular Orbitals to Be Approximately Invariant for Changes from One Molecule to Another. *Rev. Mod. Phys.* **32**, 296–299 (1960).
26. Schümann, J. M. *et al.* Exploring the Limits of Intramolecular London Dispersion Stabilization with Bulky Dispersion Energy Donors in Alkane Solution. *J. Am. Chem. Soc.* **145**, 2093–2097 (2023).
27. Baldinelli, L., De Angelis, F. & Bistoni, G. Unraveling Atomic Contributions to the London Dispersion Energy: Insights into Molecular Recognition and Reactivity. *J. Chem. Theory Comput.* **20**, 1923–1931 (2024).
28. Schümann, J. M. *et al.* Exploring the Limits of Intramolecular London Dispersion Stabilization with Bulky Dispersion Energy Donors in Alkane Solution. *J. Am. Chem. Soc.* **145**, 2093–2097 (2023).

29. Klimeš, J. & Michaelides, A. Perspective: Advances and challenges in treating van der Waals dispersion forces in density functional theory. *J. Chem. Phys.* **137**, 120901 (2012).

30. Grimme, S. Accurate description of van der Waals complexes by density functional theory including empirical corrections. *J. Comput. Chem.* **25**, 1463–1473 (2004).
