# Supplementary material for: A Quantum Chemical Method for Dissecting London Dispersion Energy into Atomic Building Blocks
Source: ACS Cent Sci. 2025 May 8;11(6):890–8. doi: 10.1021/acscentsci.5c00356 (PMC12203434; doi:10.1021/acscentsci.5c00356)
Supplement: Supplementary file 2 [file oc5c00356_si_002.pdf]

oc-2025-00356b.R1

Name: Peer Review Information for "A Quantum Chemical Method for Dissecting London Dispersion Energy into Atomic Building Blocks"

First Round of Reviewer Comments

Reviewer: 1

Comments to the Author

Referee Report on the Manuscript: 'A Quantum Chemical Method for Dissecting London Dispersion Energy into Atomic Building Blocks' by G. Regni, L. Baldinelli and G. Bistoni

The manuscript presents an Atomic Decomposition for the London Dispersion (ADLD) method. This is a novel quantum chemical approach, which the authors developed, for quantifying atomic-level contributions to London dispersion (LD) interactions. The authors combine the ADLD scheme with the Local Energy Decomposition (LED) framework at the DLPNO-CCSD(T) level, achieving new insights into dispersion interactions. The method is also combined with Grimme's DFT D3 and D4 approaches. As such, this work offers a general method that quantifies atomic contributions to LD energy with high accuracy.

The manuscript is relevant to the field of computational chemistry. It is well written, it clearly demonstrates to the reader what the authors found in their research. The authors demonstrate the method's applicability through illustrative examples.

It may be important to have such a paper published and I would recommend this paper to be published in ACS Central Science after revisions.

Let me point out the following issues, for the authors' considerations:

1. In section 2.3.3, the authors used five dimers which consist of the the same monomers and one dimer which consists of two different monomers. They found that the last dimer is

different from the gravitationally like linear fit found for the first five dimers and suggest a new expression which includes all dimers in a good linear fitting. Reviewer thinks that it is necessary to use more dimers with different monomers to be sure that the proposed equation really works for the dimers with different monomers. For example, the authors can consider a dimer which consists of dodecahedrane and tert-butane as monomers, and so on.

2. It should also be pointed out that the expression which the authors proposed cannot be considered as an equation because the expression has different units on left and right sides of the equation. The authors should revise the equation and address this issue. In any case, it would be interesting to see if their expression provides suffciently good estimations for dispersion energy based on only the masses of monomers without optimization of dimer. According to Figure 5C the dispersion energy for the proposed dimer with dodecahedrane and tert-butane as monomers should be around 45 kcal/mol. It would be interesting to compare this value to the PBE0-D4 calculated value. Furthermore, the authors should show that different methods used by them are are linearly correlated.

3. Let me also pointed out that the expression proposed by the present authors will work only for the systems where dispersion-interaction energies are considerably smaller than the dispersion energies of monomers which allows to exclude dependence on the intermonomer distances.

4. It is not completely clear which method(s) was/were used for geometry optimization of the studied systems in section 2.3. At present, one should read the Supporting information to clarify this point. Reviewer thinks that a brief note on the computational details section, may be helpful to have in the manuscript itself.

5. In the caption of the figure 5 (C) it is written; "Dependence of the total dispersion energy (E) on (M12+M22)/R while on the x-axis of figure 5C is written (M12+M22)/2". This discrepancy should be fixed.

6. In the computational details section, it is written that the cc-pVTZ basis set was employed exclusively in section 3.3. But such a section does not exist! This should be fixed.

7. Reviewer also thinks that it will be better to use the label PBE0-D4 instead of DFT-D4/PBE0.

Reviewer: 2

Comments to the Author

This article makes two contributions: (i) previously developed decomposition analysis of London dispersion is applied to two tests cases involving spin changes and size changes, (ii) a recently proposed 1/R scaling of London dispersion is examined and it is shown that this finding stems from including intramolecular dispersion effects.

From (i), I was intrigued to see how DLPNO-CCSD(T) differs from empirical D3/D4 methods.

From (ii), the decomposition of intra and intrmolecular energies is insightful and contributes to our understanding of different possible scaling laws for dispersion interactions.

Overall, the paper is well written and the analysis is well done.

One aspect that needs attention and deeper discussion is the conversion of pair terms into single-atom terms. The half/half partitioning seems rather arbitrary (and unphysical) to me. Second quantization provides a more direct way to project observables into fragments of defined size (atoms, pairs, or larger moieties). This has been done for example for the MBD method, see https://www.nature.com/articles/s41467-023-43785-z. I would like to see a deeper discussion of this fact and the connection to more advanced methodologies for dispersion interactions than empirical D3/D4 methods.

Reviewer: 3

Comments to the Author

This paper introduces a new interesting method for analyzing London dispersion interactions. The formalism has already been published elsewhere, so the emphasis here is on applications and interpretations. The latter are sometimes obvious or not well formulated yet. Greater implications are not indicated yet. This can all be addressed in a suitable revision.

For example, one of the conclusions “This observation suggests that such packing arrangements, driven by dispersion forces, are likely a common feature for apolar molecules in the condensed phase” has long been known and no new insight. What do the authors find beyond the obvious?

For the intermolecular case one wonders why the authors have only used hypothetical molecules for which no experimental data exist (like those of the Shaik analysis). Instead, the authors could have analyzed a particularly interesting case that provides an excellent playground for theory development: J. Am. Chem. Soc. 2017, 139, 7428. What is the bonding situation of the central CH••HC contact? How strong is the peripheral dispersion? Is it based on sigma-sigma or sigma-pi interactions? The system has also been analyzed in the gas phase in ACIE 2021, 60, 11305.

LD is weak for a single atom-atom interaction but adds up very quickly to become significant (often in the range of tens of kcal/mol), so calling LD generically “a weak interaction” is not quite right. This is the very reason why experimental chemists have in the past and seem to continue to ignore LD in their thinking: they consider the interaction “too weak” to be relevant to chemistry.

1,4- and 1,6-COT are not conformers but diastereomers (to be specific: valence bond isomers)! Hence, they are not in “conformational equilibrium” as the authors write in several places.

One cannot develop or use a methodology but a method.

p. 1, l. 49: even individual atom ⍰ even individual atoms

Design is by definition “rational”.

Ref. 20 and 40 are the same; it may be that 40 is supposed to be J. Am. Chem. Soc. 2023, 145, 2093.

WILEY Interdiscip. Rev. Comput. Mol. Sci. should be written as WIREs Comput. Mol. Sci.

Author's Response to Peer Review Comments:

We thank the editor for processing the manuscript and the referees for their appreciation of our work and their insightful comments. Please find enclosed a point-by-point response to the comments of the reviewers.

Reviewer 1

Recommendation: Publish in ACS Central Science after minor revisions noted.

The manuscript presents an Atomic Decomposition for the London Dispersion (ADLD) method. This is a novel quantum chemical approach, which the authors developed, for quantifying atomic-level contributions to London dispersion (LD) interactions. The authors combine the ADLD scheme with the Local Energy Decomposition (LED) framework at the DLPNO-CCSD(T) level, achieving new insights into dispersion interactions. The method is also combined with Grimme's DFT D3 and D4 approaches. As such, this work offers a general method that quantifies atomic contributions to LD energy with high accuracy.
The manuscript is relevant to the field of computational chemistry. It is well written, it clearly demonstrates to the reader what the authors found in their research. The authors demonstrate the method's applicability through illustrative examples.
It may be important to have such a paper published and I would recommend this paper to be published in ACS Central Science after revisions.

Our response: We thank the reviewer for their appreciation of our work.

Let me point out the following issues, for the authors' considerations:

1. In section 2.3.3, the authors used five dimers which consist of the the same monomers and one dimer which consists of two different monomers. They found that the last dimer is different from the gravitationally like linear fit found for the first five dimers and suggest a new expression which includes all dimers in a good linear fitting. Reviewer thinks that it is necessary to use more dimers with different monomers to be sure that the proposed equation really works for the dimers with different monomers. For example, the authors can consider a dimer which consists of dodecahedrane and tert-butane as monomers, and so on.

Our response: We thank the reviewer for the suggestion. We have extended our analysis to include additional dimers with different monomers, such as dodecahedrane and isobutane, as proposed. These new data points confirm that the proposed equation provides a good fit for a broader range of dimers.

2. It should also be pointed out that the expression which the authors proposed cannot be considered as an equation because the expression has different units on left and right sides of the equation. The authors should revise the equation and address this issue. In any case, it would be interesting to see if their expression provides suffciently good estimations for dispersion energy based on only the masses of monomers without optimization of dimer. According to Figure 5C the dispersion energy for the proposed dimer with dodecahedrane and tert-butane as monomers should be around 45 kcal/mol. It would be interesting to compare this value to the PBE0-D4 calculated value. Furthermore, the authors should show that different methods used by them are are linearly correlated.

Our response: We thank the reviewer for the comments. Our main goal in the paper was to identify a more general proportionality relationship between dispersion energy and monomers' masses using our ADLD scheme, rather than to propose a predictive equation in a strict quantitative sense. However, the identified proportionality relationship can be easily converted into a proper equation using a constant with the proper unit. This was done in the revised version.

As suggested, we have included in our study the dimer formed by dodecahedrane and isobutane. For this system, the dispersion energy computed at the PBE0-D4 level amounts to -47.3 kcal/mol, while our model predicts a value of -46.5 kcal/mol, demonstrating excellent agreement.

Finally, we would also like to clarify that the LED scheme cannot be used to compute total dispersion energies, but only inter-fragment dispersion energies. For inter-fragment dispersion, the two methods provide very similar results for systems with simple electronic structure (see Figure S12).

3. Let me also pointed out that the expression proposed by the present authors will work only for the systems where dispersion-interaction energies are considerably smaller than the dispersion energies of monomers which allows to exclude dependence on the intermonomer distances.

Our response: We agree with the reviewer's observation. By construction, our expression is expected to hold best in cases where inter-fragment dispersion energies are significantly smaller than the intra-fragment (monomer) dispersion energies. This condition is typically fulfilled in medium-sized systems, which are the main focus of our study.

4. It is not completely clear which method(s) was/were used for geometry optimization of the studied systems in section 2.3. At present, one should read the Supporting information to clarify this point. Reviewer thinks that a brief note on the computational details section, may be helpful to have in the manuscript itself.

Our response: We thank the reviewer for the helpful suggestion. A clarification regarding the methods used for the geometry optimization of the systems discussed in section 2.3 has now been added to the Computational Details section of the revised manuscript.

5. In the caption of the figure 5 (C) it is written; "Dependence of the total dispersion energy (E) on (M12+M22)/R while on the x-axis of figure 5C is written (M12+M22)/2". This discrepancy should be fixed.

Our response: We thank the reviewer for indicating this discrepancy. The incorrect expression $(M_1^2+M_2^2)/R$ in the caption of Figure 5C has been corrected.

6. In the computational details section, it is written that the cc-pVTZ basis set was employed exclusively in section 3.3. But such a section does not exist! This should be fixed.

Our response: We thank the reviewer for indicating this inconsistency. The incorrect reference to section 3.3 has been corrected.

7. Reviewer also thinks that it will be better to use the label PBE0-D4 instead of DFT-D4/PBE0.

Our response: We thank the reviewer for the suggestion. The labels have been corrected accordingly and now consistently use the notation "PBE0-D4" throughout the manuscript.

Reviewer 2

Recommendation: Publish in ACS Central Science after minor revisions noted.

Comments:
This article makes two contributions: (i) previously developed decomposition analysis of London dispersion is applied to two tests cases involving spin changes and size changes, (ii) a recently proposed 1/R scaling of London dispersion is examined and it is shown that this finding stems from including intramolecular dispersion effects.

From (i), I was intrigued to see how DLPNO-CCSD(T) differs from empirical D3/D4 methods.

From (ii), the decomposition of intra and intrmolecular energies is insightful and contributes to our understanding of different possible scaling laws for dispersion interactions.

Overall, the paper is well written and the analysis is well done.

Our response: We thank the reviewer for their appreciation of our work.

Q1. One aspect that needs attention and deeper discussion is the conversion of pair terms into single-atom terms. The half/half partitioning seems rather arbitrary (and unphysical) to me. Second quantization provides a more direct way to project observables into fragments of defined size (atoms, pairs, or larger moieties). This has been done for example for the MBD method, see https://www.nature.com/articles/s41467-023-43785-z. I would like to see a deeper discussion of this fact and the connection to more advanced methodologies for dispersion interactions than empirical D3/D4 methods.

Our response: We thank the referee for pointing us to the insightful work by Gori, Kurian, and Tkatchenko, which is now discussed in the revised manuscript. We agree that second quantization offers a well-defined framework for projecting observables onto fragments of arbitrary size.

At the same time, we note that any atomic decomposition of a non-local observable is, by nature, not unique. Different schemes - such as the one presented in our work - can provide complementary perspectives. The approach we propose is physically sound and yields chemically meaningful and consistent results across a broad range of systems, as demonstrated in this contribution.

Reviewer 3

Recommendation: Publish in ACS Central Science after minor revisions noted.

Q1. This paper introduces a new interesting method for analyzing London dispersion interactions. The formalism has already been published elsewhere, so the emphasis here is on applications and interpretations. The latter are sometimes obvious or not well formulated yet. Greater implications are not indicated yet. This can all be addressed in a suitable revision.

Our response: We thank the reviewer for their appreciation of our work. We would like to emphasize that the ADLD(LED) scheme - extending the atomic decomposition of the dispersion energy to the coupled cluster level of theory, is presented here for the first time. This extension enables a more accurate and broadly applicable analysis of dispersion interactions.

For example, one of the conclusions "This observation suggests that such packing arrangements, driven by dispersion forces, are likely a common feature for apolar molecules in the condensed phase" has long been known and no new insight. What do the authors find beyond the obvious?

Our response: We thank the reviewer for the comment. Our main goal in this section was to demonstrate the effectiveness and versatility of the method, which can find applications also in the study of molecular solids. To highlight this aspect, the sentence was rephrased as follows:

These results illustrate that the molecular packing in the solid-state is optimized to maximize dispersion interactions across all atoms. Such packing arrangements, driven by dispersion forces, are a common feature for apolar molecules in the condensed phase, and hence the ADLD scheme appears as a powerful tool to analyze the key forces that govern crystal assembly.

Q2. For the intermolecular case one wonders why the authors have only used hypothetical molecules for which no experimental data exist (like those of the Shaik analysis). Instead, the authors could have analyzed a particularly interesting case that provides an excellent playground for theory development: J. Am. Chem. Soc. 2017, 139, 7428. What is the bonding situation of the central CH••HC contact? How strong is the peripheral dispersion? Is it based on sigma-sigma or sigma-pi interactions? The system has also been analyzed in the gas phase in ACIE 2021, 60, 11305.

Our response: We thank the reviewer for bringing this very interesting system to our attention. We fully agree that it represents an excellent case study for exploring intermolecular interactions in detail. We have added references to both the *JACS* 2017 and *Angew. Chem. Int. Ed.* 2021 studies in the revised manuscript, as part of the outlook:

These findings underscore the utility of the ADLD(LED) scheme introduced here for analyzing the spatial origin of intermolecular dispersion forces. Applications of this approach to experimentally relevant systems where dispersion plays a key stabilizing role are currently underway.[41,42]

Note that the system reported in *J. Am. Chem. Soc.* 2017, *139*, 7428 consists of 214 atoms, making a thorough analysis of the bonding situation computationally demanding. To meaningfully assess the contributions of σ–σ versus σ–π interactions, coupled cluster calculations with large basis sets and TightPNO settings are required.

Q3. LD is weak for a single atom-atom interaction but adds up very quickly to become significant (often in the range of tens of kcal/mol), so calling LD generically “a weak interaction” is not quite right. This is the very reason why experimental chemists have in the past and seem to continue to ignore LD in their thinking: they consider the interaction “too weak” to be relevant to chemistry.

Our response: We clearly agree with the referee. We have revised the text to avoid reinforcing the misconception that LD is generically weak or irrelevant.

Q4. 1,4- and 1,6-COT are not conformers but diastereomers (to be specific: valence bond isomers)! Hence, they are not in “conformational equilibrium” as the authors write in several places.

Our response: We thank the reviewer for this comment. We have revised the manuscript removing any references to “conformational equilibrium” and “conformers”.

Q5. One cannot develop or use a methodology but a method.

Our response: We have replaced “methodology” with “method” in the manuscript.

Q6. p. 1, l. 49: even individual atom ◊ even individual atoms

Our response: We thank the reviewer for identifying the typo. It was corrected in the revised manuscript.

Q7. Design is by definition “rational”.

Our response: We thank the reviewer for the comment. We have revised the text for clarity.

Q8. Ref. 20 and 40 are the same; it may be that 40 is supposed to be J. Am. Chem. Soc. 2023, 145, 2093.

Our response: We have corrected the references.

Q9. WILEY Interdiscip. Rev. Comput. Mol. Sci. should be written as WIREs Comput. Mol. Sci.

Our response: We have corrected “WILEY Interdiscip. Rev. Comput. Mol. Sci.” to the appropriate abbreviation “WIREs Comput. Mol. Sci.” in the revised manuscript.
